# Heat-Killed *Lacticaseibacillus paracasei* ATG-E1 Improves Particulate Matter 10 Plus Diesel Exhaust Particles (PM_10_D)-Induced Airway Inflammation

**DOI:** 10.3390/ijms27135940

**Published:** 2026-07-01

**Authors:** Young-Sil Lee, Gun-Seok Park, Nara Jeong, Bokyeong Song, Seung-Yeon Lee, Won Ho Song, Miji Shin, Hyo-Jeong Yun, Seung-Hyun Ko, Jihee Kang

**Affiliations:** AtoGen Co., Ltd., 11-6, Techno-1 ro, Yuseong-gu, Daejeon 34015, Republic of Korea; rheeys04@atogen.co.kr (Y.-S.L.); gspark@atogen.co.kr (G.-S.P.); w41saf@atogen.co.kr (N.J.); bksong@atogen.co.kr (B.S.); yeon@atogen.co.kr (S.-Y.L.); whsong@atogen.co.kr (W.H.S.); mjshin@atogen.co.kr (M.S.); hjyun@atogen.co.kr (H.-J.Y.); ksh1229@atogen.co.kr (S.-H.K.)

**Keywords:** particulate matter, diesel exhaust particle, airway inflammation, gut microbiota, probiotics, heat-killed *L. paracasei* ATG-E1

## Abstract

Air pollutants can cause respiratory diseases, highlighting the need for effective preventive and therapeutic strategies. We investigated the protective effects of heat-killed *Lacticaseibacillus paracasei* ATG-E1 against particulate matter plus diesel exhaust particle (PM_10_D)-induced airway inflammation. BALB/c mice were intranasally injected with PM_10_D and treated with heat-killed *L. paracasei* ATG-E1 via oral gavage for 5 days. In the bronchoalveolar lavage fluid (BALF) and lungs, inflammatory mediators, immune cell subtypes, and histological changes were analyzed, while gut microbiota composition was analyzed in the cecum. Heat-killed *L. paracasei* ATG-E1 suppressed the infiltration of immune cells, including neutrophils, T cells, and B cells. Furthermore, it decreased various inflammatory mediators, such as C-X-C Motif chemokine ligand (CXCL)-1, macrophage inflammatory protein (MIP)-2, interleukin (IL)-1α, and tumor necrosis factor (TNF)-α, in the BALF and lung tissue, as well as serum symmetric dimethylarginine (SDMA) levels in the PM_10_D-induced airway inflammation model. Heat-killed *L. paracasei* ATG-E1 also exhibited a protective effect against lung damage induced by PM_10_D. Furthermore, heat-killed *L. paracasei* ATG-E1 treatment shifted the gut microbiota composition, increasing several bacterial genera. The data demonstrate that heat-killed *L. paracasei* ATG-E1 acts as a protective agent against air pollutant-induced lung injury, suggesting its potential as a candidate adjunctive strategy for prevention.

## 1. Introduction

Rapid industrial development has caused environmental air pollution, which is associated with serious and harmful effects on human health worldwide [1]. According to the World Health Organization (WHO), in 2019, 99% of the world’s population was exposed to air pollutants, exceeding the WHO guidelines, and global estimates revealed 4.2 million premature deaths annually due to ambient air pollution [2]. Air pollutants comprise gaseous and particulate matter (PM) [3]. PM is a complex mixture of solid and liquid particles generated by fuel combustion, industrial emissions, and road and agricultural dust and contains chemical constituents, such as metals, elemental and organic carbon, sulfates, nitrates, organic compounds, and biological compounds [4,5]. PMs are classified based on their aerodynamic diameter as follows: PM_10_ (<10 μm, coarse particles), PM_2.5_ (<2.5 μm, fine particles), or PM_0.1_ (<0.1 μm, ultrafine particulates). PMs less than PM_10_ have the greatest impact on human health [6,7]. Upon contact with PM, epithelial and inflammatory cells, such as alveolar macrophages (AMs), are activated and produce inflammatory mediators, including TNF-α, IL-1, and IL-6, which contribute to local inflammatory responses in the lungs [8]. Beyond acute inflammation, chronic PM_10_ exposure promotes epithelial injury, oxidative stress, and persistent inflammatory signaling, leading to activation of pro-fibrotic pathways such as transforming growth factor (TGF)-β and STAT3/ERK. These processes drive epithelial-to-mesenchymal transition, extracellular matrix deposition, subepithelial fibrosis, and goblet cell hyperplasia, thereby contributing to airway remodeling [9]. Furthermore, it may induce the development of pulmonary diseases and exacerbate pre-existing ones, including respiratory infection, asthma, chronic obstructive pulmonary disease (COPD), lung cancer, heart disease, and premature mortality [10]. On a global scale, this individual pathology translates into a profound disease burden, driving a disproportionate increase in morbidity, healthcare costs, and disability-adjusted life years specifically for obstructive airway diseases. In particular, recent global estimates indicate that up to 41.27% of the total attributable risk for COPD is directly related to PM exposure [11]. For these reasons, there is a growing interest in novel preventive and therapeutic agents that can protect against PM-induced respiratory diseases.

Probiotics are well-recognized for their immunomodulatory, anti-obesity, and metabolic benefits. Emerging evidence further suggests that they provide beneficial effects against respiratory diseases, such as respiratory tract infection, asthma, cystic fibrosis, COPD, and lung cancer, as well as in PM-induced lung inflammation and injury [12,13]. Therefore, probiotics could be an alternative target to alleviate respiratory diseases. Although probiotics confer health benefits to the host, some safety concerns remain regarding the use of live probiotic strains in the food and pharmaceutical industries. To address these concerns, research interest in inactivated bacterial cells (parabiotics) and their metabolites (postbiotics) is gradually increasing [14]. The use of inactivated probiotics has advantages over live probiotics, including enhanced safety and greater stability for long-term storage. According to many reports, heat-killed probiotics can provide various health benefits similar to live probiotics, such as immune modulation and gut microbiota modulation. However, the bioactivity of a specific strain cannot be generalized, as the functional components (e.g., cell wall constituents or metabolites) and their stability during the inactivation process are highly strain dependent [14,15,16]. We previously demonstrated that viable *Lacticaseibacillus paracasei* ATG-E1 effectively mitigates airway inflammation when administered prophylactically before PM_10_ plus diesel exhaust particle (PM_10_D) exposure [17]. Building on these findings, the present study evaluated the protective potential of heat-killed *L. paracasei* ATG-E1 against PM_10_D-induced airway inflammation using a modified administration schedule, where heat-killed *L. paracasei* ATG-E1 began 3 days after the onset of the PM_10_D challenge. This approach allows for an independent assessment of whether the strain maintains its protective capacity when introduced after the respiratory insult has already commenced, thereby addressing the stability and safety limitations of live bacteria under a distinct exposure timeline.

## 2. Results

### 2.1. Effects of Heat-Killed L. paracasei ATG-E1 on Lung Tissue Damage

**Figure 1:**
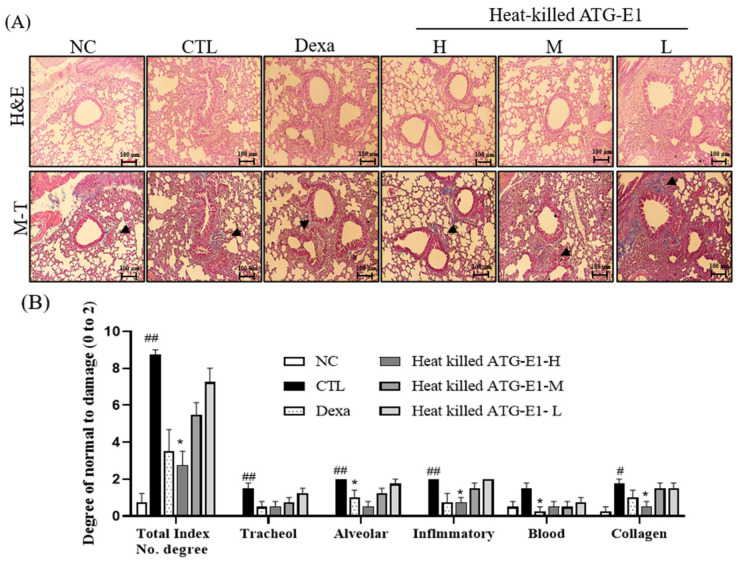
Effects of heat-killed *L. paracasei* ATG-E1 on histological changes in a PM_10_D-induced airway inflammation animal model. (**A**) H&E and MT staining of lung tissues and (**B**) quantitative analysis of lung tissue damage. H&E staining was performed to evaluate histopathological changes and inflammatory cell infiltration, while MT staining was utilized to assess collagen deposition and tissue fibrosis in the peribronchiolar regions. Scale bar = 100 μm. Black arrows indicate administered particulate matter (PM_10_D). NC: BALB/c normal mice; CTL: PM_10_D-induced control mice; Dexa: dexamethasone (3 mg/kg)-treated PM_10_D-induced mice; H: 1 × 10^10^ heat-killed *L. paracasei* ATG-E1 cell-treated PM_10_D-induced mice; M: 1 × 10^9^ heat-killed *L. paracasei* ATG-E1 cell-treated PM_10_D-induced mice; L: 1 × 10^8^ heat-killed *L. paracasei* ATG-E1 cell-treated PM_10_D-induced mice. Data are expressed as mean ± SEM (*n* = 8). ^#^ *p* < 0.05 and ^##^ *p* < 0.01 vs. NC; * *p* < 0.05 vs. CTL.

PM exposure caused histopathological changes in lung tissue. To assess the protective effects of heat-killed *L. paracasei* ATG-E1 against lung tissue damage, mice were administered with heat-killed *L. paracasei* ATG-E1, followed by PM_10_D intranasal injection. As shown in Figure 1, histological changes, such as inflammation around the bronchus, collagen deposition, destruction of alveolar cells, and mucus in respiratory epithelial cells, were more frequent in the PM_10_D-induced control (CTL) group than in the normal (NC) group. These histological changes in lung tissue were protected by treatment with heat-killed *L. paracasei* ATG-E1-H (high-dose).

### 2.2. Effects of Heat-Killed L. paracasei ATG-E1 on the Total Number of Cells and Neutrophils in BALF

**Figure 2:**
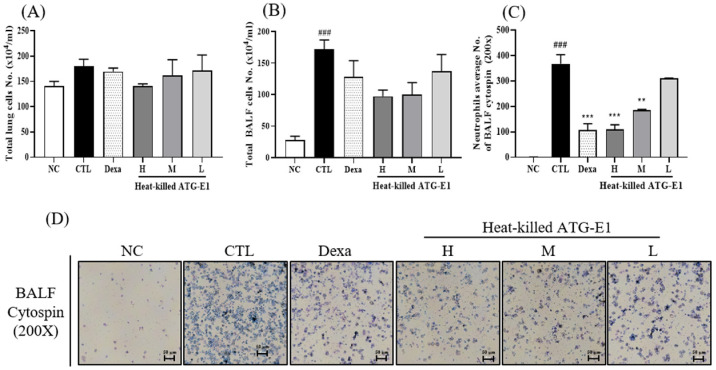
Effects of heat-killed *L. paracasei* ATG-E1 on airway cell numbers in a PM_10_D-induced airway inflammation animal model. (**A**) Total lung cell number and (**B**) total cell number in bronchoalveolar lavage fluid (BALF) were quantified using a hemocytometer. (**C**) Differential neutrophil counts were determined from BALF cytospin preparations. (**D**) Representative photomicrographs of BALF cytospin slides (×200; scale bar = 50 μm) stained to visualize the infiltration of inflammatory cells, such as neutrophils and macrophages, into the airway. NC: BALB/c normal mice; CTL: PM_10_D-induced control mice; Dexa: dexamethasone (3 mg/kg)-treated PM_10_D-induced mice; H: PM_10_D-induced mice treated with 1 × 10^10^ heat-killed *L. paracasei* ATG-E1 cells; M: PM_10_D-induced mice treated with 1 × 10^9^ heat-killed *L. paracasei* ATG-E1 cells; L: PM_10_D-induced mice treated with 1 × 10^8^ heat-killed *L. paracasei* ATG-E1 cells. Data are expressed as mean ± SEM (*n* = 8). ^###^ *p* < 0.005 vs. NC; ** *p* < 0.01, and *** *p* < 0.005 vs. CTL.

Lung damage caused by PMs was affected by airway inflammation through the infiltration of various immune cells and inflammatory responses. No significant difference in the total lung number was observed among the treated groups (Figure 2A). The total BALF cell numbers decreased in the NC and heat-killed *L. paracasei* ATG-E1-H groups compared with the CTL group; however, this number did not change in the dexamethasone, heat-killed *L. paracasei* ATG-E1-M (middle dose), and L (low dose) groups (Figure 2B). The neutrophil infiltration into the BALF significantly decreased in all treated groups, except for the heat-killed *L. paracasei* ATG-E1-L group, compared with the CTL group (Figure 2C,D).

### 2.3. Effects of Heat-Killed L. paracasei ATG-E1 on BALF and Lung Immune Cell Populations

Given the observed reduction in total immune cells and neutrophils, flow cytometry was utilized to characterize specific leukocyte subsets. In BALF (Table 1), lymphocyte counts were largely unaffected by treatment. Eosinophils/macrophages and CD62L^−^CD44^+high^ cells decreased in the NC group relative to the CTL group, which was not observed in the ATG-E1-treated groups. However, both the NC and ATG-E1-H groups exhibited a significant decrease in neutrophils and Gr-1^+^SiglecF^−^ cells. Furthermore, Gr-1^+^CD11b^+^ cells were lower in the NC, heat-killed *L. paracasei* ATG-E1-H, and ATG-E1-M groups than in the CTL group. In the lungs, the absolute number of lymphocytes, neutrophils, eosinophils/macrophages, and CD4^+^ cells did not exhibit significant changes among the treated groups, though a distinct difference in neutrophils existed between the NC and CTL groups. CD8^+^ cells decreased in the NC and dexamethasone groups. Notably, CD62L^−^CD44^+high^ cells were decreased in all treated groups except for the heat-killed *L. paracasei* ATG-E1-L group, whereas CD21/35^+^B220^+^ cells Gr-1^+^CD11b^+^ and Gr-1^+^SiglecF^−^ cells were reduced in the dexamethasone and heat-killed *L. paracasei* ATG-E1-H groups compared with the CTL group.

### 2.4. Effects of Heat-Killed L. paracasei ATG-E1 on the Levels of Inflammatory Mediators in BALF and SDMA Levels in the Serum

**Figure 3:**
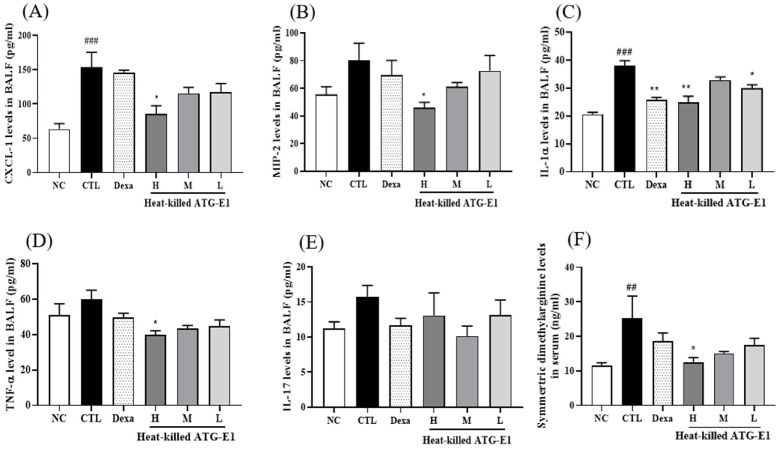
Effects of heat-killed *L. paracasei* ATG-E1 on inflammatory mediators in BALF and SDMA levels in the serum of a PM_10_D-induced airway inflammation animal model. The concentrations of proinflammatory chemokines, (**A**) CXCL-1 and (**B**) MIP-2, and cytokines, (**C**) IL-1α, (**D**) TNF-α, and (**E**) IL-17, in the bronchoalveolar lavage fluid (BALF) were quantified by ELISA. (**F**) Serum levels of symmetric dimethylarginine (SDMA) were measured as a systemic biomarker. NC: BALB/c normal mice; CTL: PM_10_D-induced control mice; Dexa: dexamethasone (3 mg/kg)-treated PM_10_D-induced mice; H: PM_10_D-induced mice treated with 1 × 10^10^ heat-killed *L. paracasei* ATG-E1 cells; M: PM_10_D-induced mice treated with 1 × 10^9^ heat-killed *L. paracasei* ATG-E1 cells; L: PM_10_D-induced mice treated with 1 × 10^8^ heat-killed *L. paracasei* ATG-E1 cells. Data are expressed as mean ± SEM (*n* = 8). ^##^ *p* < 0.01 and ^###^ *p* < 0.005 vs. NC; * *p* < 0.05 and ** *p* < 0.01 vs. CTL.

Compared with the CTL group, lower CXCL-1 and MIP-2 levels were observed in the NC and heat-killed *L. paracasei* ATG-E1-H groups (Figure 3A,B). Except for the ATG-E1-M group, all treatments led to a decline in BALF IL-1α levels in BALF (Figure 3C). TNF-α levels were significantly reduced in the heat-killed *L. paracasei* ATG-E1-H group (Figure 3D), whereas IL-17A levels remained unchanged among the groups (Figure 3E). Furthermore, serum SDMA levels were significantly lower in the NC and heat-killed *L. paracasei* ATG-E1-H groups than in the CTL group (Figure 3F).

### 2.5. Effects of Heat-Killed L. paracasei ATG-E1 on the Expression Levels of Inflammatory Mediators in the Lungs

**Figure 4:**
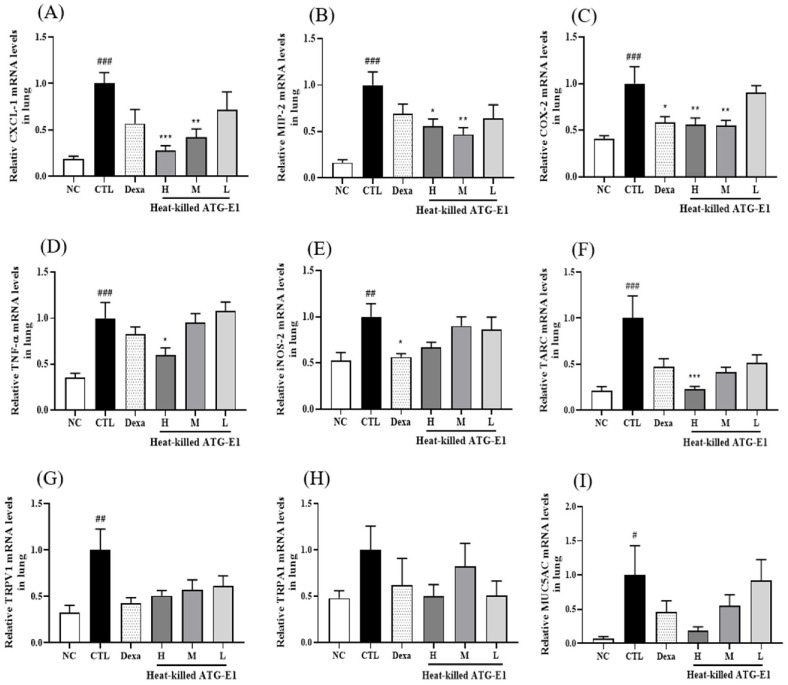
Effects of heat-killed *L. paracasei* ATG-E1 on the pulmonary expression of inflammatory mediators and related genes in a PM_10_D-induced airway inflammation animal model. The mRNA expression levels of proinflammatory mediators, including chemokines (**A**) *CXCL-1* and (**B**) *MIP-2*, inflammatory enzymes (**C**) *COX-2* and (**E**) *iNOS-2*, and a cytokine (**D**) *TNF-α*, alongside cough- and pain-related sensory channels (**F**) *TRAC*, (**G**) *TRPV1*, and (**H**) *TRPA1* and a mucin gene (**I**) *MUC5AC*, were quantified in lung tissues using RT-qPCR. NC: BALB/c normal mice; CTL: PM_10_D-induced control mice; Dexa: dexamethasone (3 mg/kg)-treated PM_10_D-induced mice; H: PM_10_D-induced mice treated with 1 × 10^10^ heat-killed *L. paracasei* ATG-E1 cells; M: PM_10_D-induced mice treated with 1 × 10^9^ heat-killed *L. paracasei* ATG-E1 cells; L: PM_10_D-induced mice treated with 1 × 10^8^ heat-killed *L. paracasei* ATG-E1 cells. Data are expressed as mean ± SEM (*n* = 8). ^#^ *p* < 0.05, ^##^ *p* < 0.01, and ^###^ *p* < 0.005 vs. NC; * *p* < 0.05, ** *p* < 0.01, and *** *p* < 0.005 vs. CTL.

Compared with the CTL group, a notable reduction in *CXCL-1*, *MIP-2*, and *COX-2* mRNA expression in the lungs was observed following treatment with the NC, heat-killed *L. paracasei* ATG-E1-H, and M groups, whereas dexamethasone also suppressed *COX-2* expression (Figure 4A–C). *TNF-α* expression was significantly diminished only in the NC and heat-killed *L. paracasei* ATG-E1-H groups (Figure 4D), whereas *iNOS-2* expression was selectively reduced in the NC and dexamethasone groups (Figure 4E). Notably, thymus and activation-regulated chemokine expression was downregulated in the heat-killed *L. paracasei* ATG-E1-H groups (Figure 4F). Among sensory receptor-related genes, transient receptor potential vanilloid (TRPV)1 and transient Receptor Potential Ankyri (TRPA)1 mRNA expression was unaffected by treatment (Figure 4G,H). Mucin 5, subtypes A and C, tracheobronchial/gastric (*MUC5AC*) was significantly suppressed in the NC group and showed a non-significant tendency toward reduction in the heat-killed *L. paracasei* ATG-E1-H group (Figure 4I).

### 2.6. Transcriptome Changes Induced by Heat-Killed L. paracasei ATG-E1 in the Lungs

**Figure 5:**
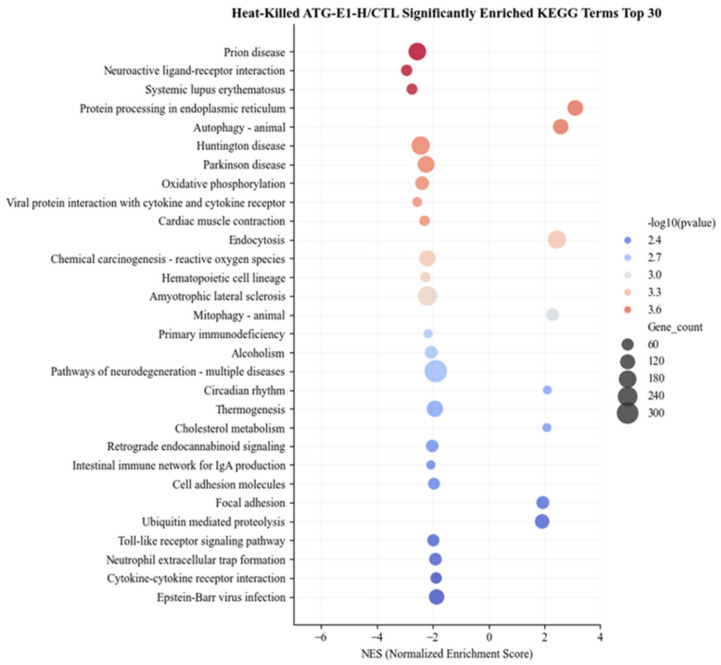
KEGG GSEA results. KEGG GSEA was performed using transcriptomic data from the heat-killed *L. paracasei* ATG-E1-H and CTL groups. The top 30 enriched pathways were selected based on *p*-values and are presented in the figure. The x-axis represents the normalized enrichment score, whereas the y-axis displays the KEGG pathway names. Bubble size corresponds to the number of genes associated with each pathway, and bubble color represents the −log10(*p*-value).

To explore transcriptomic changes associated with the anti-inflammatory effects of heat-killed *L. paracasei* ATG-E1, RNA sequencing was performed using lung tissue samples. Each RNA-seq sample consisted of pooled lung tissues from two mice, and three biological sequencing samples were analyzed per group. The detailed RNA-seq workflow, including quality control, read trimming, mapping, normalization, and differentially expressed gene (DEG) selection criteria, is described in the Supplementary Materials and Methods. DEGs were selected using a cutoff of |fold change| ≥ 2 and raw *p* < 0.05. In the heat-killed *L. paracasei* ATG-E1-H-treated group compared with the PM_10_D-sensitized control group, 71 DEGs were identified, including 21 upregulated and 50 downregulated genes (Supplementary Figure S1). KEGG gene set enrichment analysis (GSEA) was then performed to identify pathways associated with the transcriptomic changes. The top 30 KEGG pathway terms were selected based on *p*-values and are presented in Figure 5. In the lungs of the *L. paracasei* ATG-E1-H-treated group, enriched pathways included the Toll-like receptor (TLR) signaling pathway, neutrophil extracellular trap (NET) formation, and cytokine–cytokine receptor interaction (Supplementary Figure S2), which are related to inflammatory responses.

### 2.7. Effects of Heat-Killed L. paracasei ATG-E1 on the Inflammatory Pathway in the Lungs

**Figure 6:**
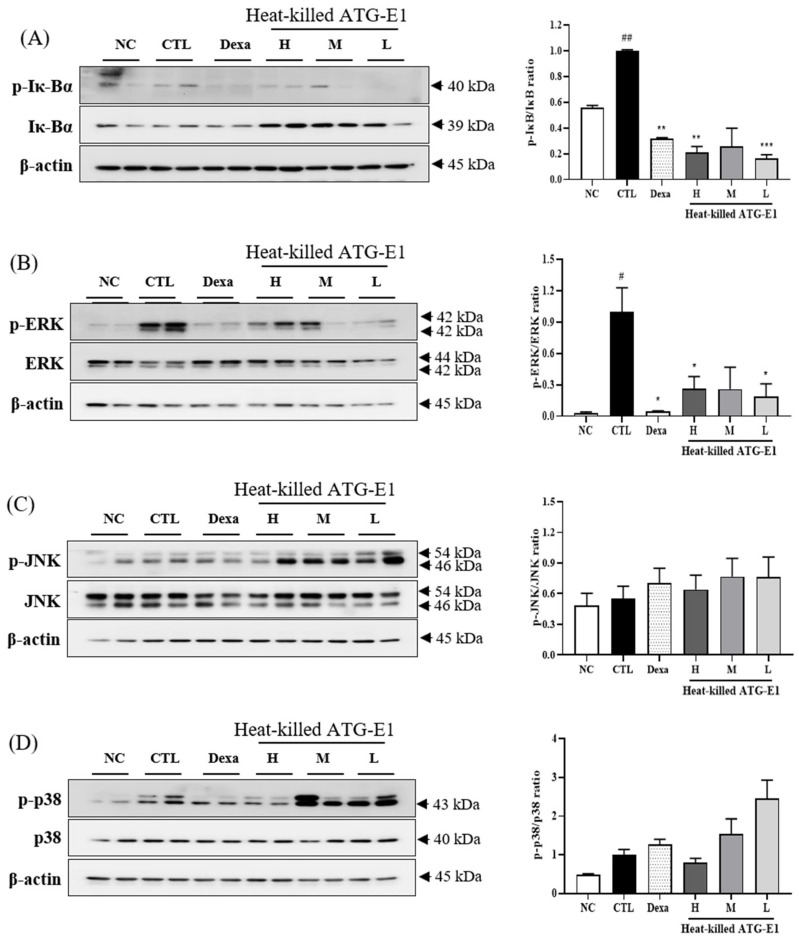
Effects of heat-killed *L. paracasei* ATG-E1 on inflammatory signaling pathways in the lungs. Phosphorylation and densitometric quantification of (**A**) IκBα, (**B**) ERK, (**C**) JNK, and (**D**) p38 MAPK in lung tissues. Western blotting and densitometric analyses were performed to assess the phosphorylation status of these signaling molecules. NC: BALB/c normal mice; CTL: PM_10_D-induced control mice; Dexa: dexamethasone (3 mg/kg)-treated PM_10_D-induced mice; H: PM_10_D-induced mice treated with 1 × 10^10^ heat-killed *L. paracasei* ATG-E1 cells; M: PM_10_D-induced mice treated with 1 × 10^9^ heat-killed *L. paracasei* ATG-E1 cells; L: PM_10_D-induced mice treated with 1 × 10^8^ heat-killed *L. paracasei* ATG-E1 cells. Data are expressed as mean ± SEM (*n* = 8). ^#^ *p* < 0.05 and ^##^ *p* < 0.01 vs. NC; * *p* < 0.05, ** *p* < 0.01, and *** *p* < 0.005 vs. CTL.

**Figure 7:**
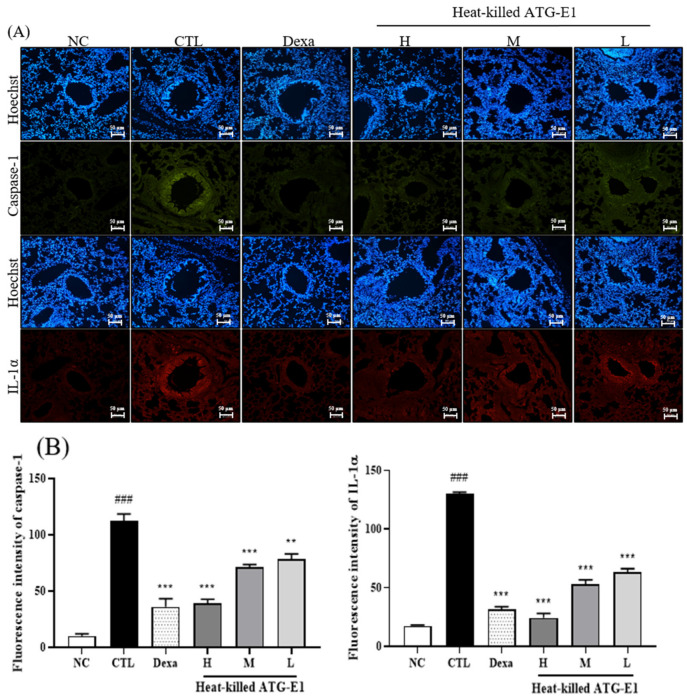
Effects of heat-killed *L. paracasei* ATG-E1 on caspase-1 and IL-1α expression in the lung tissues of a PM_10_D-induced airway inflammation animal model. Representative immunofluorescence images of lung tissues stained for (**A**) caspase-1 and IL-1α (Scale bar = 50 μm). Densitometric quantification of (**B**) caspase-1 and IL-1α. Nuclei were counterstained with Hoechst 33342, and fluorescence intensity was quantified via densitometric analysis using ImageJ. NC: BALB/c normal mice; CTL: PM_10_D-induced control mice; Dexa: dexamethasone (3 mg/kg)-treated PM_10_D-induced mice; H: PM_10_D-induced mice treated with 1 × 10^10^ heat-killed *L. paracasei* ATG-E1 cells; M: PM_10_D-induced mice treated with 1 × 10^9^ heat-killed *L. paracasei* ATG-E1 cells; L: PM_10_D-induced mice treated with 1 × 10^8^ heat-killed *L. paracasei* ATG-E1 cells. Data are expressed as mean ± SEM (*n* = 8). ^###^ *p* < 0.005 vs. NC; ** *p* < 0.01 and *** *p* < 0.005 vs. CTL.

PMs and diesel exhaust particles (DEPs) are recognized by TLRs, which are responsible for the induction of innate and adaptive immune responses. This recognition sequentially triggers the activation of downstream signaling molecules, including myeloid differentiation primary response 88 (MyD88), TIR domain-containing adapter protein (TIRAP), interleukin-1 receptor-associated kinase (IRAK), and TNF receptor-associated factor 6 (TRAF6), thereby initiating inflammatory signaling cascades. Subsequently, they can activate the NF-κB and MAPK pathways, which induce inflammatory mediators [18]. The total protein expression of Iκ-Bα significantly increased, and the phosphorylation of Iκ-Bα and ERK was inhibited, but not JNK and p38, in the heat-killed *L. paracasei* ATG-E1 and dexamethasone-treated groups compared with those in the CTL group (Figure 6). In the transcriptome analysis of the lungs, heat-killed *L. paracasei* ATG-E1-H tended to exhibit reduced expression of TLR/MyD88/TIRAP/IRAK1/TRAF6, an upstream pathway of NF-κB and MAPK. Furthermore, PMs and DEPs can induce immune cell death and excessive inflammatory responses. Next, we examined the effects of heat-killed *L. paracasei* ATG-E1 on cell death. As shown in Figure 7, the fluorescence intensity of caspase-1 and IL-1α in the lungs was reduced in the NC, dexamethasone, and heat-killed *L. paracasei* ATG-E1-treated groups compared with that in the CTL group.

### 2.8. Effects of Heat-Killed L. paracasei ATG-E1 on Cecal Microbiota Composition

**Figure 8:**
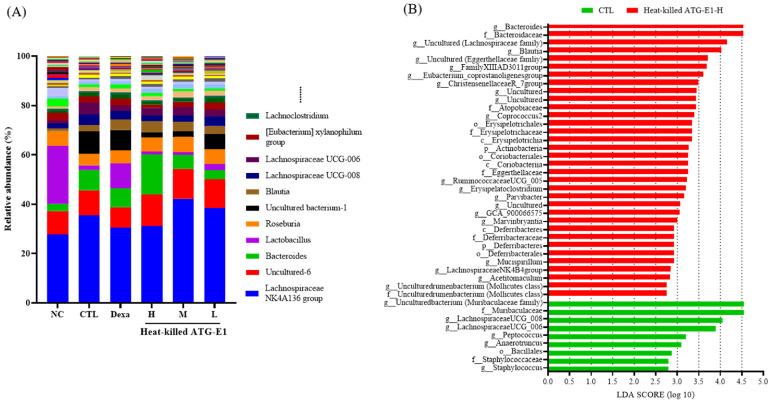
Effects of heat-killed *L. paracasei* ATG-E1 on cecal microbiota composition and taxonomic profiles. (**A**) Relative abundance (%) of bacterial genera. (**B**) Linear discriminant analysis effect size (LEfSe) analysis showing differentially abundant taxa between the CTL and heat-killed *L. paracasei* ATG-E1-treated groups. Taxa with LDA scores (log10) > 2.0 and *p* < 0.05 are shown. NC: BALB/c normal mice; CTL: PM_10_D-induced control mice; Dexa: dexamethasone (3 mg/kg)-treated PM_10_D-induced mice; H: PM_10_D-induced mice treated with 1 × 10^10^ heat-killed *L. paracasei* ATG-E1 cells; M: PM_10_D-induced mice treated with 1 × 10^9^ heat-killed *L. paracasei* ATG-E1 cells; L: PM_10_D-induced mice treated with 1 × 10^8^ heat-killed *L. paracasei* ATG-E1 cells. Data are expressed as mean ± SEM (*n* = 8).

Figure 8A shows the relative abundance of each sample at the genus level. The most common genus was the Lachnospiraceae NK4A136 group. The *Lactobacillus* genus showed a relative abundance of 23.4% in the NC group, whereas the other groups showed a relative abundance of <10%. To further investigate the differences in taxonomic abundance between the CTL group with PM_10_D-induced airway inflammation and the dexamethasone- or ATG-E1-H-treated group, linear discriminant analysis effect size (LEfSe) analysis was performed. Figure 8B presents a histogram of the linear discriminant analysis (LDA) value distribution, indicating taxa that were significantly abundant in either group. *Acetitomaculum*, *Mucispirillum*, *Marvinbryantia*, *Parvibacter*, *Erysipelatoclostridium*, *Blautia*, and *Bacteroides* were significantly abundant in the high-dose heat-killed *L. paracasei* ATG-E1-H-treated group. To further investigate the impact of heat-killed *L. paracasei* ATG-E1 on the microbial ecosystem, we performed additional α- and β-diversity and correlation analyses. In α-diversity, the Shannon index did not show marked differences among groups, whereas the Chao1 estimator was significantly increased in the heat-killed *L. paracasei* ATG-E1-H group compared the CTL group. Principal coordinate analysis based on Bray–Curtis dissimilarity was also performed to evaluate β-diversity. Pairwise PERMANOVA analysis showed that PM_10_D exposure significantly altered the gut microbial community structure compared with the NC group, and that heat-killed *L. paracasei* ATG-E1-H treatment significantly shifted the community structure compared with the CTL group (Supplementary Figure S3A–C). Next, based on the LEfSe results and specific taxa identified in the prior microbiota analysis, we performed exploratory Spearman correlation analyses between the selected gut bacterial taxa and BALF inflammatory mediators. In particular, selected taxa, including *Marvinbryantia* and *Parvibacter*, showed negative correlations with BALF IL-1α levels (Supplementary Figure S3D,E).

## 3. Discussion

Air pollutants (PMs and DEPs) trigger proinflammatory immune responses that affect various immune cell types, such as AMs, neutrophils, and dendritic cells, which can orchestrate complex adaptive immune responses in the respiratory tract [19]. As first-responder immune cells, AMs and neutrophils can respond rapidly through phagocytosis upon inhalation of PMs and DEP. Upon activation by PMs and DEP, the TLR-MyD88/IRAK/TAK-1 signaling pathways can activate the NF-κB and MAPK pathways, which in turn stimulate proinflammatory responses, such as TNF-α and IL-6 [10,18,20]. Furthermore, they can induce cell death, resulting in excessive inflammatory responses. Caspase-1-dependent pyroptosis, a form of cell death, is associated with cell death [10]. Inhaled particulates induce alveolar macrophage cell death, IL-1α secretion, and subsequent formation of inducible bronchus-associated lymphoid tissue, which is involved in the inflammation response in the lungs [21,22]. In our study, heat-killed *L. paracasei* ATG-E1 not only decreased the number of various immune cells, including infiltrated neutrophils, Gr-1^+^SiglecF^−^, Gr-1^+^CD11b^+^, CD62L^−^CD44^high+^, and CD21/35^+^B220^+^ cells but also inhibited the expression of various inflammatory mediators, such as CXCL-1, MIP-2, IL-1α, TNF-α, COX-2, iNOS-2, TRAC, TRPV1, and TRPA1, in the BALF and lungs of the PM_10_D-induced airway inflammation model. Similarly, in the KEGG mapper of the transcriptome analysis of the lung, cytokines, such as TNF-α and IL-1β, and cytokine–cytokine receptor interactions, such as the CC (CCL2 and CCL12) and CXC (CXCL5 and CXCL6) subfamilies, were reduced by heat-killed *L. paracasei* ATG-E1. Next, we found that heat-killed *L. paracasei* ATG-E1 reduced or tended to reduce the expression of various genes associated with the TLR signaling pathway, including TLR1/2/6, MyD88, TRAF6, IKAK1, and AP-1, based on the KEGG mapper of the transcriptome analysis of the lung. Simultaneously, heat-killed *L. paracasei* ATG-E1 reduced cell death by inhibiting caspase-1 activation and IL-1α expression. Heat-killed *L. paracasei* ATG-E1 suppressed ERK and IκBα phosphorylation, whereas JNK and p38 phosphorylation remained unchanged in the lungs of the PM_10_D-induced airway inflammation model. In our in vivo model, because the JNK and p38 pathways were not initially stimulated by PM_10_D, the lack of suppressive effect on JNK and p38 by the killed *L. paracasei* ATG-E1 is a reasonable and consistent outcome. Taken together, these results imply that the protective effects of the killed *L. paracasei* ATG-E1 bacteria may be closely associated with the downregulation of the PM-responsive inflammatory axes, the Iκ-Bα and ERK pathways, in our study. Nevertheless, since direct mechanism validations including p65 nuclear translocation and receptor inhibitor assays were not performed, our findings remain largely associated. Further in vitro studies using macrophage or epithelial cell lines are needed to fully clarify the exact molecular mechanisms.

From the histological data, we observed that heat-killed *L. paracasei* ATG-E1 exhibits protective effects against lung tissue damage, represented by neutrophil infiltration, collagen and mucin accumulation, and alveolar cell destruction, in the airway. With reduced airway inflammation by heat-killed *L. paracasei* ATG-E1, these results seem to be associated with a reduction in NET formation in the KEGG mapper of the transcriptome analysis of the lung, MUC5AC expression, and serum SDMA levels. NETs, a meshwork of inflammatory molecules released from neutrophils, are an effective defense against pathogens; however, the excessive formation and release of NETs (NETosis) can induce direct tissue damage and infiltration of other proinflammatory cells and cytokines [23]. SDMA, a competitor of the transporter for L-arginine, may reduce the production of nitric oxide (NO), which contributes to vascular smooth muscle relaxation and mucus secretion in the airway [24]. MUC5AC also increases mucus production and airway obstruction [25]. These results indicate that heat-killed *L. paracasei* ATG-E1 protects against PM_10_D-induced airway remodeling. Although RNA-seq identified key inflammatory pathways —TLR signaling, NET formation, and cytokine–cytokine receptor interaction—only a subset of representative genes was validated via qPCR. Further validation of a broader gene panel remains a subject for future studies.

By promoting a healthy gut microbiota, probiotics can indirectly and directly benefit respiratory health, offering a promising approach for managing conditions influenced by the gut–lung axis [26,27,28,29]. Based on our findings, administration of heat-killed *L. paracasei* ATG-E1 increased the abundance of several specific bacterial taxa, including *Acetitomaculum*, *Mucispirillum*, *Marvinbryantia*, *Parvibacter*, *Erysipelatoclostridium*, *Blautia*, and *Bacteroides*. Regarding the structural profile of the intestinal microbiota, this treatment successfully replenished microbial richness—as demonstrated by an elevated Chao1 index—and driven a distinct shift in β-diversity. Interestingly, exploratory Spearman correlation analysis indicated that pulmonary inflammatory markers were dictated by a specific cluster of ATG-E1-responsive microbes rather than overall community alterations. Notably, Marvinbryantia and Parvibacter, which were markedly enriched following ATG-E1 treatment, exhibited strong inverse correlations with BALF IL-1α levels. Research on the aforementioned genus anti-inflammatory effect is limited; however, a few studies have investigated the anti-inflammatory properties of those genera. Previous studies have reported that *Parvibacter* may attenuate inflammation by enhancing the expression of the tight junction protein ZO-1, thereby reducing the systemic translocation of lipopolysaccharides (LPS) [30]. More recently, *Marvinbryantia* (specifically *M. formatexigens*) was shown to suppress intestinal inflammation by stimulating short-chain fatty acid (SCFA) production, which subsequently fortifies the gut epithelial barrier [31]. Additionally, *Acetitomaculum* is an acetate-producing genus that generates metabolites capable of supporting neighboring microbial communities and maintaining intestinal barrier integrity [32]. These findings may be relevant to our observation that heat-killed *L. paracasei* ATG-E1 significantly upregulated the expression of several tight junction genes, including *occludin*, *claudin-1*, *claudin-4*, and *claudin-5* (Supplementary Figure S4). Furthermore, *Bacteroides* produces capsular polysaccharide A (PSA), which promotes regulatory T (Treg) cell differentiation and IL-10 production through interactions with dendritic cells, thereby contributing to immune homeostasis [33]. In addition to modulating the gut microbiota and intestinal barrier function, ATG-E1 treatment also influenced local intestinal immune responses. Heat-killed *L. paracasei* ATG-E1 tended to decrease the absolute numbers of CD4^+^, CD8^+^, and CD4^+^CD69^+^ cells in Peyer’s patch (Supplementary Figure S5). Given that Peyer’s patches represent a pivotal inductive site for mucosal immunity, the suppression of these activated T-cell subsets indicates that heat-killed *L. paracasei* ATG-E1 may effectively counteracts localized immunoinflammatory cascades. Crucially, these microbial and immunomodulatory shifts were accompanied by distinct alterations in the functional output of the microbiota. While the gut microbiota typically produces major SCFAs (such as butyric, propionic, and acetic acids) to modulate host immunity and barrier function [26], cecal levels of these primary SCFAs remained unchanged following heat-killed *L. paracasei* ATG-E1 treatment. Instead, specific branched-chain fatty acids (BCFAs), such as isobutyric and isovaleric acids levels, were higher after heat-killed *L. paracasei* ATG-E1 treatment. Although research on this topic is limited, increased isobutyric and isovaleric acid levels have been observed in mice with inflammatory bowel disease, in which the disease was alleviated [34]. These results suggest that the alterations in BCFAs by heat-killed *L. paracasei* ATG-E1 might potentially modulate immune and inflammatory responses in the gut. Taken together, these results suggest that heat-killed *L. paracasei* ATG-E1 treatment is associated with changes in gut microbiota composition, gut immune responses, gut barrier-related gene expression, and microbial metabolites, which may be linked to reduced inflammatory responses in the lung. However, because the present study did not directly test microbiota-dependent causality, further studies using microbiota transfer, antibiotic depletion, or metabolite validation approaches are needed to determine whether these gut microbiota changes directly contribute to airway protection.

In this study, heat-killed *L. paracasei* ATG-E1 demonstrated a dose-dependent profile. The high dose consistently produced robust anti-inflammatory effects, whereas the middle and low doses showed weak, inconsistent responses. This dose dependency carries strong biological plausibility given the pharmacological nature of inactivated biologics. Unlike live *L. paracasei* ATG-E1 that proliferates in vivo, the efficacy of the heat-killed formulation depends on initial concentration delivered to the target site. Thus, while the high dose successfully overcomes host clearance to trigger anti-inflammatory pathways, lower and middle doses are rapidly cleared or degraded, failing to initiate or sustain meaningful downstream signaling cascades. These results confirm that a definitive Minimum Effective Dose (MED) lies between the middle and high doses tested. Crucially, this high dose represents a clinically realistic and safe regimen, as it induced no demonstrable cytotoxicity or adverse systemic effects. Nonetheless, subsequent dose-finding studies narrowing the interval between the middle and high doses will be essential to define the exact MED and clarify the therapeutic safety margin of ATG-E1.

While this study demonstrated the protective potential of heat-killed *L. paracasei* ATG-E1 against PM_10_D-induced airway inflammation, several limitations should be addressed in future studies to further elucidate the underlying mechanisms. First, the gut-derived mediators responsible for systemic signaling to the lungs, such as specific microbial metabolites or migrating immune cell subsets, remain to be identified. Second, this study did not include a non-specific bacterial control, such as a heat-killed strain from a different genus; thus, we cannot entirely rule out baseline immunostimulatory effects inherent to common bacterial cell wall components like peptidoglycans. Future comparative studies incorporating such controls are warranted to definitively confirm the strain specificity of *L. paracasei* ATG-E1. Third, concerns may arise regarding the potential immunostimulatory effects of the aluminum hydroxide vehicle used in the PM_10_D model. While the robust neutrophil response in our control (CTL) group may appear more pronounced than that reported in some adjuvant-free models, this 1% vehicle was administered uniformly to all groups, including the normal control. Therefore, any potential baseline immunomodulatory effects of the vehicle were equally distributed across groups and are not expected to confound the interpretation of PM_10_D-induced airway inflammation. Forth, although this study primarily focused on the protective effects of the heat-killed form, evaluating its efficacy relative to its live counterpart provides important context for its potential utility. To address this, we confirmed that the anti-inflammatory efficacy of heat-killed *L. paracasei* ATG-E1 was comparable to that of the live bacteria, with no significant difference observed in the attenuation of airway inflammation (Supplementary Figure S6). While these comparable phenotypic effects are promising, comprehensive follow-up studies aimed at elucidating the precise molecular signaling pathways and distinct cellular interactions of the live and heat-killed forms remain warranted to fully clarify their respective mechanisms of action.

There have been concerns regarding whether the beneficial effects of probiotics depend on the unique properties of live bacteria. However, our parallel evaluation of the two forms (Supplementary Figure S6) demonstrates that heat inactivation does not compromise the strain’s intrinsic biological activity. This is consistent with a substantial body of published evidence showing that nonviable probiotics can also exert robust biological effects, including modulation of intestinal homeostasis and immune responses [16]. Crucially, while our previous report underscored the benefits of live *L. paracasei* ATG-E1 administered prior to PM_10_D exposure [17], the current study extends this line of research by confirming its protective capacity under a different intervention window. Specifically, administration of heat-killed *L. paracasei* ATG-E1 beginning three days after PM_10_D exposure demonstrated that heat inactivation does not impair the strain’s ability to modulate ongoing inflammatory responses. Given that the heat-killed form maintains efficacy comparable to its live counterpart while eliminating the risk of bacterial translocation and improving manufacturing stability, these findings represent an important step toward safer and more versatile industrial applications.

## 4. Materials and Methods

### 4.1. Preparation of Heat-Killed L. paracasei ATG-E1 Samples

To prepare heat-killed *L. paracasei* ATG-E1 (KCTC 14245BP), the strain was cultured in MRS broth at 37 °C for 16 h and subsequently heat-inactivated at 90 °C for 30 min. The resulting suspension was centrifuged at 3000× *g* for 10 min at 4 °C, and the pellets were washed three times with phosphate-buffered saline (PBS; pH 7.4). To confirm complete bacterial inactivation, the heat-treated suspension was plated onto MRS agar. The collected cell pellets were then mixed with a cryoprotectant and lyophilized using an IlShinBioBase freeze-dryer (Model FD8508, Seoul, Republic of Korea).

### 4.2. Animals and Experimental Scheme

Male BALB/c mice, aged 6 weeks, were sourced from Orient Bio Co., Ltd. (Seongnam-si, Republic of Korea) and housed under standard laboratory conditions (22 °C ± 2 °C, 60% ± 10% humidity, and a 12 h light/dark cycle) with unrestricted access to food and water. All experimental procedures followed the guidelines authorized by the Institutional Animal Care and Use Committee of Daejeon University (DJUARB2021-025, Issued on 10 June 2021). To minimize the use of experimental animals in accordance with ethical guidelines, the control groups (NC, CTL, dexamethasone) in this study were shared with a parallel investigation evaluating the effects of live *L. paracasei* ATG-E1. Following a 1-week stabilization period, the mice received a mixture of PM (aerodynamic diameter < 10 μm; Sigma-Aldrich, St. Louis, MO, USA) and DEP (Sigma-Aldrich) prepared in 1% aluminum hydroxide gel adjuvant via intranasal administration on days 0, 3, and 6. Concurrently, a 5-day oral administration of heat-killed *L. paracasei* ATG-E1 was initiated on day 3, representing 3 days post-initial PM_10_D treatment (Supplementary Figure S7). The experimental design comprised seven groups (*n* = 8 mice/group): (1) NC, 1% aluminum hydroxide gel adjuvant- and vehicle-treated mice; (2) PM_10_D_CTL, challenged with PM_10_+DEP and given vehicle; (3) PM_10_D_Dexa, treated with dexamethasone (3 mg/kg) as a positive control; (4) PM_10_D_ATG-E1-H, treated with 1 × 10^10^ cells of heat-killed *L. paracasei* ATG-E1; (5) PM_10_D_ATG-E1-M, treated with 1 × 10^9^ cells of heat-killed *L. paracasei* ATG-E1; and (6) PM_10_D_ATG-E1-L, treated with 1 × 10^8^ cells of heat-killed *L. paracasei* ATG-E1. The freeze-dried heat-killed *L. paracasei* ATG-E1 powder was resuspended in saline and prepared daily for the animal experiment periods. For the NC and CTL groups, the vehicle consisting of the identical lyophilized protectant matrix (without bacteria) was suspended in saline and prepared daily to serve as an equivalent carrier control during the animal experiment periods.

### 4.3. Flow Cytometric Analysis of Bronchoalveolar Lavage Fluid (BALF) and Lung Tissues

Isolation procedures for lung and BALF cells are detailed in the Supplementary Materials and Methods. To conduct flow cytometric analysis, single-cell suspensions obtained from these tissues were stained with specific monoclonal antibodies against various surface markers: FITC-conjugated anti-CD4 (clone RM4-5), PE-conjugated anti-CD8a (53-6.7), PE-conjugated anti-CD62L (MEL-14), PE-Cy5.5-conjugated anti-CD44 (IM7), PE-conjugated anti-Gr-1 (RB6-8C5), FITC-conjugated anti-CD11b (M1/70), PE-Cy7-conjugated anti-SiglecF (1RNM44N), FITC-conjugated anti-CD21/CD35 (7G6), and PE-conjugated anti-B220 (RA3-6B2). Nonviable cells were ruled out utilizing 7-AAD positivity. After fixation with 0.5% paraformaldehyde, fluorescence signals were acquired on a FACSCalibur flow cytometer equipped with CellQuest software v5.2.1 (BD Biosciences, Mountain View, CA, USA). Multiparameter data analysis and fluorescence compensation were subsequently performed using FlowJo^TM^ software v10 (licensed via dongle). Further details regarding the experimental methods are described in the Supplementary Materials and Methods.

### 4.4. BALF Cytological Analysis

BALF-isolated cells were deposited onto glass slides using a cytocentrifuge (800 rpm, 5 min) and subsequently processed with a Diff-Quick Stain kit (Baxter Healthcare Corp., Miami, FL, USA), after which neutrophil numbers were quantified. Further details regarding the experimental methods are described in the Supplementary Materials and Methods.

### 4.5. Measurement of Various Inflammatory Mediators in BALF and Symmetric Dimethylarginine (SDAM) in the Serum

BALF chemokines (MIP-2 and CXCL-1) and cytokines (IL-1α, TNF-α, and IL-17A) were quantified using commercial kits from R&D Systems (St. Louis, MO, USA). Serum SDAM concentrations were also determined (MYBioSource.com, San Diego, CA, USA). All procedures were performed according to protocols provided by each manufacturer.

### 4.6. RNA Isolation and Gene Expression Analysis

Total RNA from lung tissue was isolated using TRIzol^®^ reagent (Thermo-Fisher Scientific, Waltham, MA, USA). Subsequently, complementary DNA (cDNA) was synthesized with the AccuPower RT PreMix (Bioneer, Daejeon, Republic of Korea) following the provided protocol. Quantitative polymerase chain reaction (qPCR) analyses were then performed on an Applied Biosystems 7500 Real-Time PCR system v2.3, employing SYBR Green PCR Master Mix (Applied Biosystems, Foster City, CA, USA) to evaluate gene expression levels. The specific primer sequences utilized in this study are detailed in Supplementary Table S1.

### 4.7. Western Blotting

Lung total proteins were isolated in radioimmunoprecipitation assay lysis buffer (Millipore, Germany) in the presence of Xpert Duo Inhibitor Cocktail Solution (GenDEPOT, Katy, TX, USA), and homogenized via a Tissuelyser II (Qiagen, Hilden, Germany). The extracted proteins were separated on Mini-PROTEAN^®^ TGX^TM^ Precast Protein Gels (Bio-Rad, Hercules, CA, USA) before being transferred to PVDF membranes using a Trans-Blot^®^ Turbo^TM^ Transfer System with the Trans-Blot Turbo RTA Transfer kit (Bio-Rad, Hercules, CA, USA). Following a blocking step with skim milk (BD Biosciences, San Diego, CA, USA), primary antibodies specific for p-ERK, ERK, p-JNK, JNK, p-p38, p38, p-IκBα, IκBα, and β-actin (Cell Signaling Technology, Danvers, MA, USA) and secondary antibodies (Bio-Rad, Hercules, CA, USA) were applied to the membranes. Chemiluminescent signals were developed using Clarity Max Western ECL Substrate (Bio-Rad, Hercules, CA, USA), detected with a ChemiDoc^TM^ Touch Gel Imaging System (Bio-Rad, Hercules, CA, USA), and quantified through Image Lab^TM^ software (v6.0; Bio-Rad, Hercules, CA, USA).

### 4.8. Histological Analysis

Lung tissue specimens were fixed in a formalin solution, embedded in paraffin wax, and subsequently sectioned at a thickness of 5 μm. Staining of the lung sections was performed using hematoxylin–eosin (H&E) and Masson’s trichrome (M-T). For quantitative histological analysis, random fields were selected from each specimen. A semi-quantitative grading system was utilized to score various pathological features, including inflammatory cell infiltration, hemorrhage, tracheolar/alveolar changes, collagen deposition, and goblet cell hyperplasia. Each feature was evaluated on a 0–2 scale by two independent, blinded observers [35].

### 4.9. Immunofluorescence Staining

Lung sections (20 µm thick) underwent fixation with 4% paraformaldehyde supplemented with sucrose in PBS for 40 min, followed by permeabilization using 0.5% Nonidet P-40 (Sigma-Aldrich, St. Louis, MO, USA) in PBS. To prevent non-specific binding, the tissue sections were blocked with 2.5% horse serum and bovine serum albumin. For immunofluorescence analysis, the tissue sections were incubated overnight at 4 °C with primary antibodies targeting caspase-1 and IL-1α. After washing, a fluorescein-conjugated secondary antibody was applied, and nuclei were visualized by counterstaining with Hoechst 33342. Fluorescent signals were captured with a fluorescence microscope (Nikon Instruments, Inc., Mississauga, ON, Canada) and analyzed quantitatively through ImageJ software v1.54.

### 4.10. Transcriptome Analysis

Lung tissues were immediately stabilized in RNAlater solution (Invitrogen, Waltham, MA, USA) and subsequently transferred to Macrogen Inc. (Seoul, Republic of Korea) for library preparation using the TruSeq Stranded Total RNA LT Sample Prep Kit (Gold; Illumina, San Diego, CA, USA). RNA sequencing was in accordance with a previously reported study [18]. Each RNA-seq sample consisted of pooled lung tissues from two mice, and three biological sequencing samples were analyzed per group; detailed sequencing quality metrics, read mapping statistics, DEG selection criteria, and pathway analysis procedures are provided in the Supplementary Materials and Methods. Quality control of the generated raw sequencing reads was conducted via FastQC (v. 0.11.7), and adapter sequences were removed using Trimmomatic (v. 0.38) [36]. The processed sequences were aligned to the reference genome (mm10) using HISAT2 (v. 2.1.0). Transcript assembly and quantification were performed with StringTie (v. 2.1.3b) based on the reference annotation model [37]. Each sample was normalized using DESeq2 v1.42.0 of the R package, and fold changes and *p*-values were calculated for each comparison. These data were used to identify statistically significant DEGs [38]. All raw RNA sequencing data generated in this study have been deposited in the NCBI Sequence Read Archive (SRA) database under BioProject accession number PRJNA1009041. Comprehensive procedural details are available in the Supplementary Materials and Methods.

### 4.11. Cecal Microbiota Analysis

Genomic DNA was isolated from cecal microbial cells using the PowerFecal DNA Isolation Kit (Mo Bio Laboratories, Carlsbad, CA, USA) following the manufacturer’s instructions. To evaluate the concentration and integrity of the yielded DNA, a Qubit Flex Fluorometer (Thermo-Fisher Scientific, Waltham, MA, USA) and a LabChip GX Touch HT (PerkinElmer, Waltham, MA, USA) were employed, respectively. Amplification of the V3–V4 hypervariable regions of the bacterial 16S rRNA gene was followed as previously reported [39]. The resulting raw sequence data were uploaded to the NCBI SRA repository (BioProject: PRJNA100878). Subsequent bioinformatics processing of the raw reads was performed using the QIIME pipeline [40], where quality-filtered sequences were clustered into operational taxonomic units (OTUs) based on a 97% similarity threshold against the SILVA database (version 138.1) [41]. Taxonomic classification of OTUs was determined at the phylum and genus levels. Differential abundance analysis was conducted using linear discriminant analysis effect size (LEfSe), and features with an LDA score > 2.0 and a false discovery rate (FDR)-adjusted *p*-value < 0.1 were considered statistically significant. Additional methodological details are provided in the Supplementary Materials and Methods Section [42,43,44].

### 4.12. Statistical Analysis

All data are reported as means ± SEM. Prior to conducting parametric analyses, we validated the assumptions of normality and equal variance using the Shapiro–Wilk and Brown–Forsythe test. Group means were compared using a one-way ANOVA, and Dunnett’s test was applied as a post hoc analysis to evaluate differences between the control and treated groups. For data that did not follow a normal distribution, the Kruskal–Wallis test followed by Dunn’s post hoc test was utilized. In cases where the assumption of normality was met but equal variance was violated, Welch’s ANOVA followed by Dunnett’s T3 post hoc test was performed. For bacterial community analyses, one-way ANOVA followed by Tukey’s post hoc test or an unpaired two-tailed *t*-test was used, as appropriate. For β-diversity analysis, pairwise PERMANOVA was performed based on Bray–Curtis dissimilarity, and adjusted *p*-values were used to account for multiple comparisons. For exploratory microbiome–cytokine associations, Spearman correlation analysis was performed, and false discovery rate-adjusted q-values were calculated. For the statistical analysis of the transcriptome results, read count data were normalized using relative log expression size factors in the DESeq2 R package. Differential expression analysis was performed using the negative binomial Wald test (nbinomWaldTest), and DEGs were selected using |fold change| ≥ 2 and raw *p* < 0.05. GSEA was performed using the modified Fisher’s exact test to evaluate the significance of KEGG pathways generated from the transcriptome results. GraphPad Prism version 7.0 (GraphPad Software, San Diego, CA, USA) was used for all statistical analyses.

## 5. Conclusions

Heat-killed *L. paracasei* ATG-E1 inhibited immune and inflammatory responses, protected against lung tissue damage, and regulated the gut microbiome in a PM_10_D-induced airway inflammation model. The respiratory-improving effects of heat-killed *L. paracasei* ATG-E1 are potentially mediated by the interaction between the gut and lungs, indicating that modulating the gut microbiota could play a crucial role in managing respiratory health. These findings suggest that heat-killed *L. paracasei* ATG-E1 may serve as a potential adjunctive strategy for PM-induced airway inflammation, accompanied by local suppression of lung inflammation and concomitant changes in the gut microbiota.

## Figures and Tables

**Table 1:** Effects of heat-killed *L. paracasei* ATG-E1 on airway immune cell numbers in a PM_10_D-induced airway inflammation model (absolute counts).

Cell Phenotype					Heat-Killed *L. paracasei* ATG-E1		
		NC	CTL	Dexa	H	M	L
Lymphocytes (×10^4^ cells)	BALF	2.07 ± 0.59	4.39 ± 0.95	2.54 ± 0.87	11.04 ± 4.16	6.20 ± 0.50	8.23 ± 1.71
Neutrophils (×10^4^ cells)		4.85 ± 1.52	92.42 ± 11.69 ^###^	62.26 ± 13.16	24.86 ± 7.46 **	43.74 ± 8.62 *	59.88 ± 15.04
Eosinophils/Macrophage (×10^4^ cells)		15.25 ± 7.50	67.01 ± 4.19 ^##^	44.95 ± 8.08	47.42 ± 6.08	45.92 ± 10.17	63.54 ± 9.54
CD62L^−^CD44^+high^ (×10^4^ cells)		1.33 ± 1.14	110.07 ± 23.57 ^#^	82.02 ± 18.65	62.87 ± 11.05	65.92 ± 14.44	76.39 ± 9.59
Gr-1^+^SiglecF^−^ (×10^4^ cells)		0.38 ± 0.14	63.23 ± 6.71 ^###^	43.03 ± 12.43	16.65 ± 6.08 **	32.62 ± 3.75	39.63 ± 11.99
Gr-1^+^CD11b^+^ (×10^7^ cells)		0.70 ± 0.22	98.96 ± 6.65 ^###^	62.96 ± 14.82	29.75 ± 8.13 **	49.26 ± 7.70 *	62.19 ± 18.55
Lymphocytes (×10^4^ cells)	Lung	78.04 ± 45.90	38.92 ± 6.33	45.48 ± 2.48	32.40 ± 0.90	34.00 ± 7.69	42.84 ± 11.57
Neutrophils (×10^7^ cells)		28.71 ± 14.93	101.43 ± 14.28 ^##^	71.07 ± 4.76	60.84 ± 2.75	85.88 ± 18.54	87.70 ± 15.00
Eosinophils/Macrophage (×10^4^ cells)		16.60 ± 8.46	36.50 ± 2.28	49.06 ± 4.92	58.41 ± 10.78	39.06 ± 5.61	38.25 ± 6.78
CD4^+^ (×10^4^ cells)		34.27 ± 17.67	78.33 ± 10.34	63.40 ± 4.33	52.11 ± 2.20	65.65 ± 14.06	72.50 ± 12.57
CD8^+^ (×10^4^ cells)		7.09 ± 2.37	67.59 ± 14.73 ^##^	27.32 ± 6.54 *	41.15 ± 2.47	37.53 ± 9.87	38.55 ± 4.78
CD62L^−^CD44^+high^ (×10^4^ cells)		11.71 ± 1.69	566.34 ± 139.59 ^###^	149.30 ± 38.55 **	285.57 ± 36.39 *	247.69 ± 36.35 *	321.79 ± 44.12
CD21/35^+^B220^+^ (×10^4^ cells)		9.26 ± 2.88	282.33 ± 41.83 ^###^	70.79 ± 14.83 ***	94.05 ± 10.15 *	105.10 ± 31.28	151.80 ± 17.78
Gr-1^+^CD11b^+^ (×10^4^ cells)		12.69 ± 6.55	70.21 ± 12.55 ^##^	45.69 ± 3.68 *	39.11 ± 2.09 *	46.53 ± 11.30	71.46 ± 14.54
Gr-1^+^SiglecF^−^ (×10^4^ cells)		6.52 ± 3.30	47.02 ± 11.35 ^##^	19.97 ± 2.59 *	19.01 ± 2.92 *	25.08 ± 5.80	46.99 ± 8.27

NC: BALB/c normal mice; CTL: PM_10_D-induced control mice; Dexa: dexamethasone (3 mg/kg)-treated PM_10_D-induced mice; H: PM_10_D-induced mice treated with 1 × 10^10^ heat-killed *L. paracasei* ATG-E1 cells; M: PM_10_D-induced mice treated with 1 × 10^9^ heat-killed *L. paracasei* ATG-E1 cells; L: PM_10_D-induced mice treated with 1 × 10^8^ heat-killed *L. paracasei* ATG-E1 cells. ^#^ *p* < 0.05, ^##^ *p* < 0.01 and ^###^ *p* < 0.005 vs. NC; * *p* < 0.05, ** *p* < 0.01, and *** *p* < 0.005 vs. CTL.

## Data Availability

The datasets generated and/or analyzed during this study are available at the NCBI’s repository. The raw sequence data of bacterial community sequencing were submitted to the NCBI’s SRA database (NCBI BioProject PRJNA1008786, https://www.ncbi.nlm.nih.gov/bioproject/1008786 (accessed on 23 June 2026)). The raw sequencing data of the transcriptome analysis discussed in this publication are deposited in the NCBI’s SRA database (NCBI BioProject PRJNA PRJNA1009041, http://www.ncbi.nlm.nih.gov/bioproject/1009041 (accessed on 23 June 2026).

## Supplementary Materials

The following supporting information can be downloaded at: https://www.mdpi.com/article/10.3390/ijms27135940/s1.
