# Supplementary material for: Heat-Killed Lacticaseibacillus paracasei ATG-E1 Improves Particulate Matter 10 Plus Diesel Exhaust Particles (PM10D)-Induced Airway Inflammation
Source: Int J Mol Sci. 2026 Jul 1;27(13):5940. doi: 10.3390/ijms27135940 (PMC13361942; doi:10.3390/ijms27135940)
Supplement: Supplementary file 1 [file ijms-27-05940-s001.zip › Supplementary Figures and Table.pptx]

## Slide 1
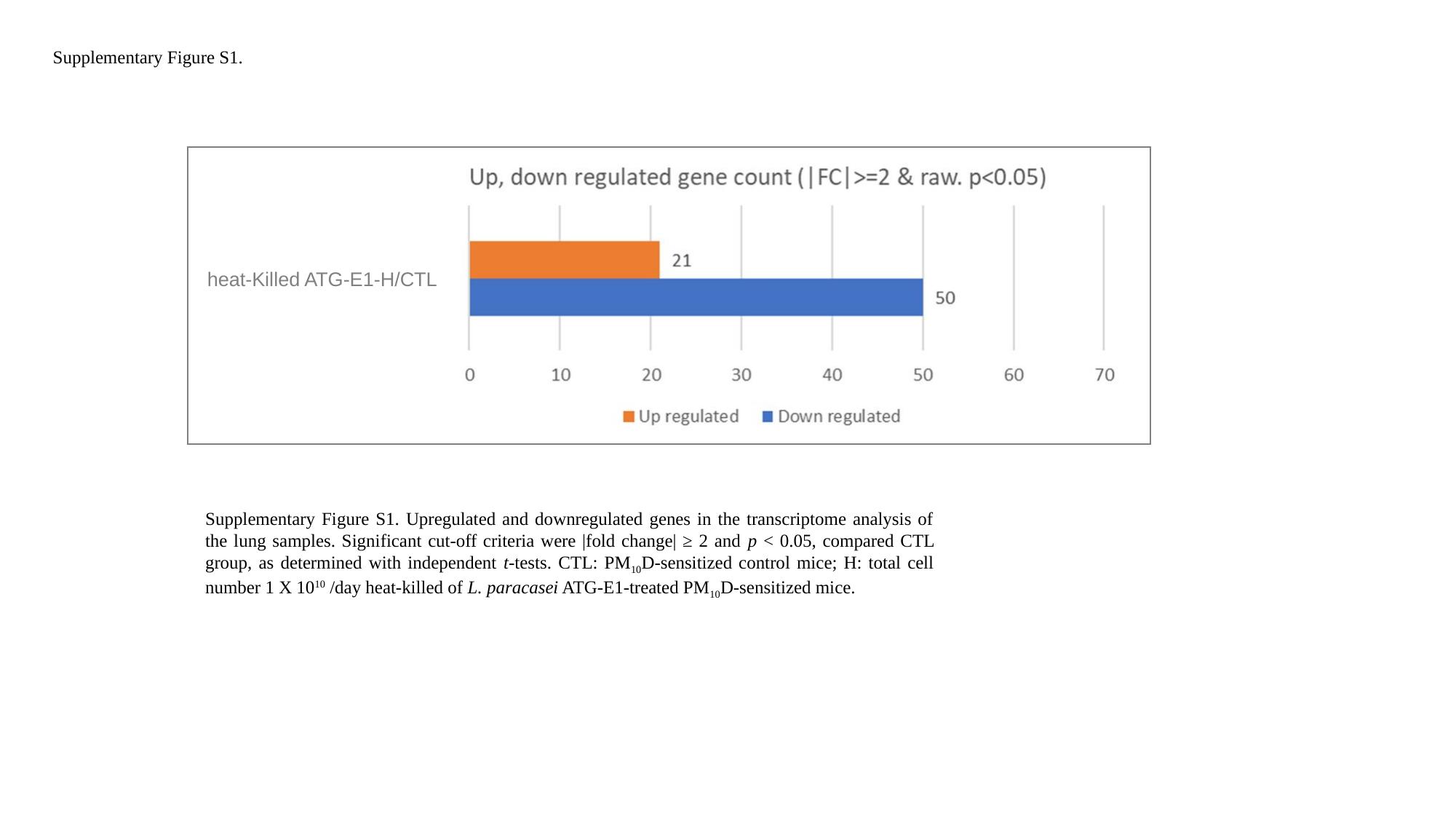

Supplementary Figure S1.
heat-Killed ATG-E1-H/CTL
Supplementary Figure S1. Upregulated and downregulated genes in the transcriptome analysis of the lung samples. Significant cut-off criteria were |fold change| ≥ 2 and p < 0.05, compared CTL group, as determined with independent t-tests. CTL: PM10D-sensitized control mice; H: total cell number 1 X 1010 /day heat-killed of L. paracasei ATG-E1-treated PM10D-sensitized mice.

## Slide 2
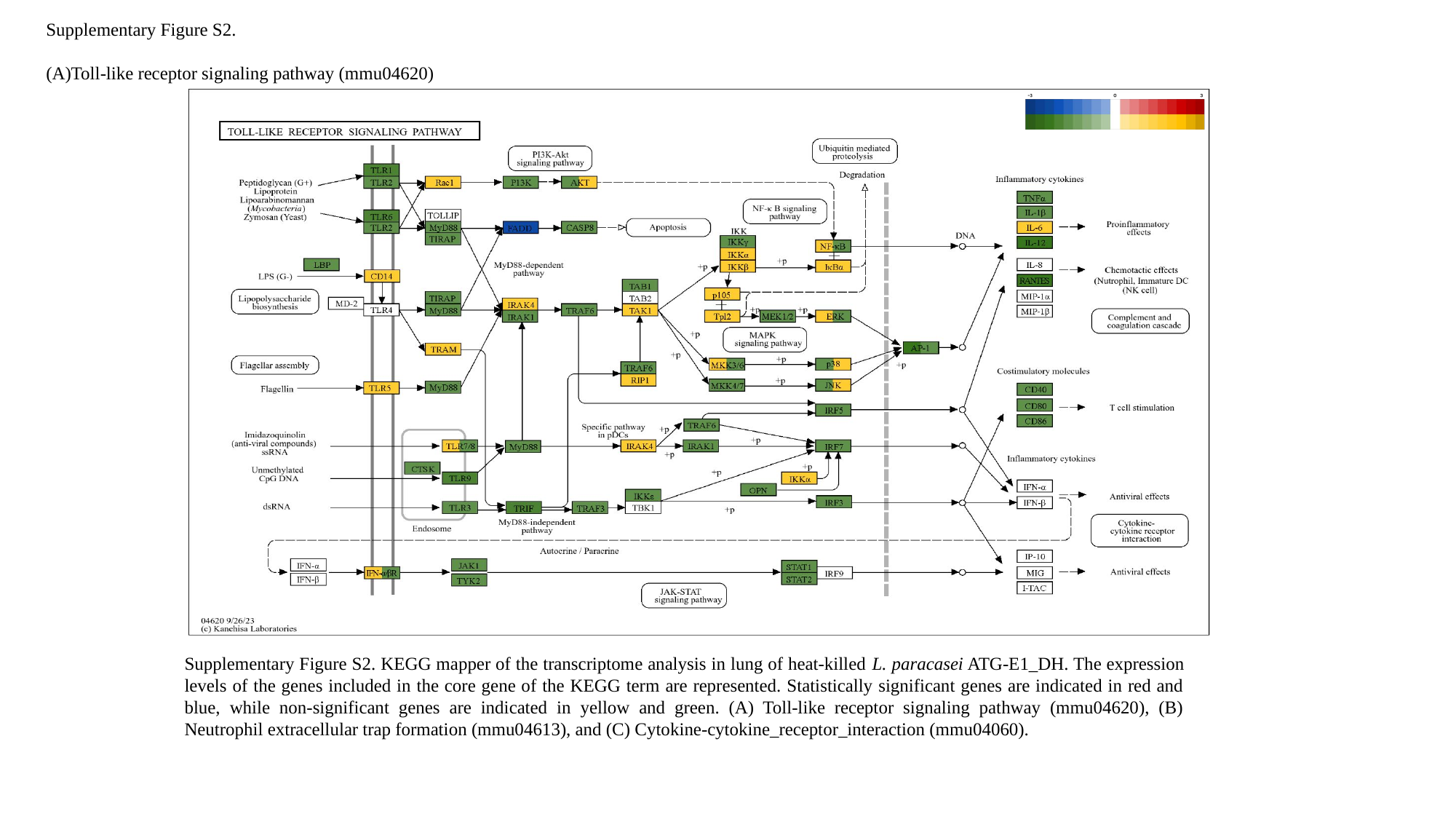

Supplementary Figure S2.
(A)Toll-like receptor signaling pathway (mmu04620)
Supplementary Figure S2. KEGG mapper of the transcriptome analysis in lung of heat-killed L. paracasei ATG-E1_DH. The expression levels of the genes included in the core gene of the KEGG term are represented. Statistically significant genes are indicated in red and blue, while non-significant genes are indicated in yellow and green. (A) Toll-like receptor signaling pathway (mmu04620), (B) Neutrophil extracellular trap formation (mmu04613), and (C) Cytokine-cytokine_receptor_interaction (mmu04060).

## Slide 3
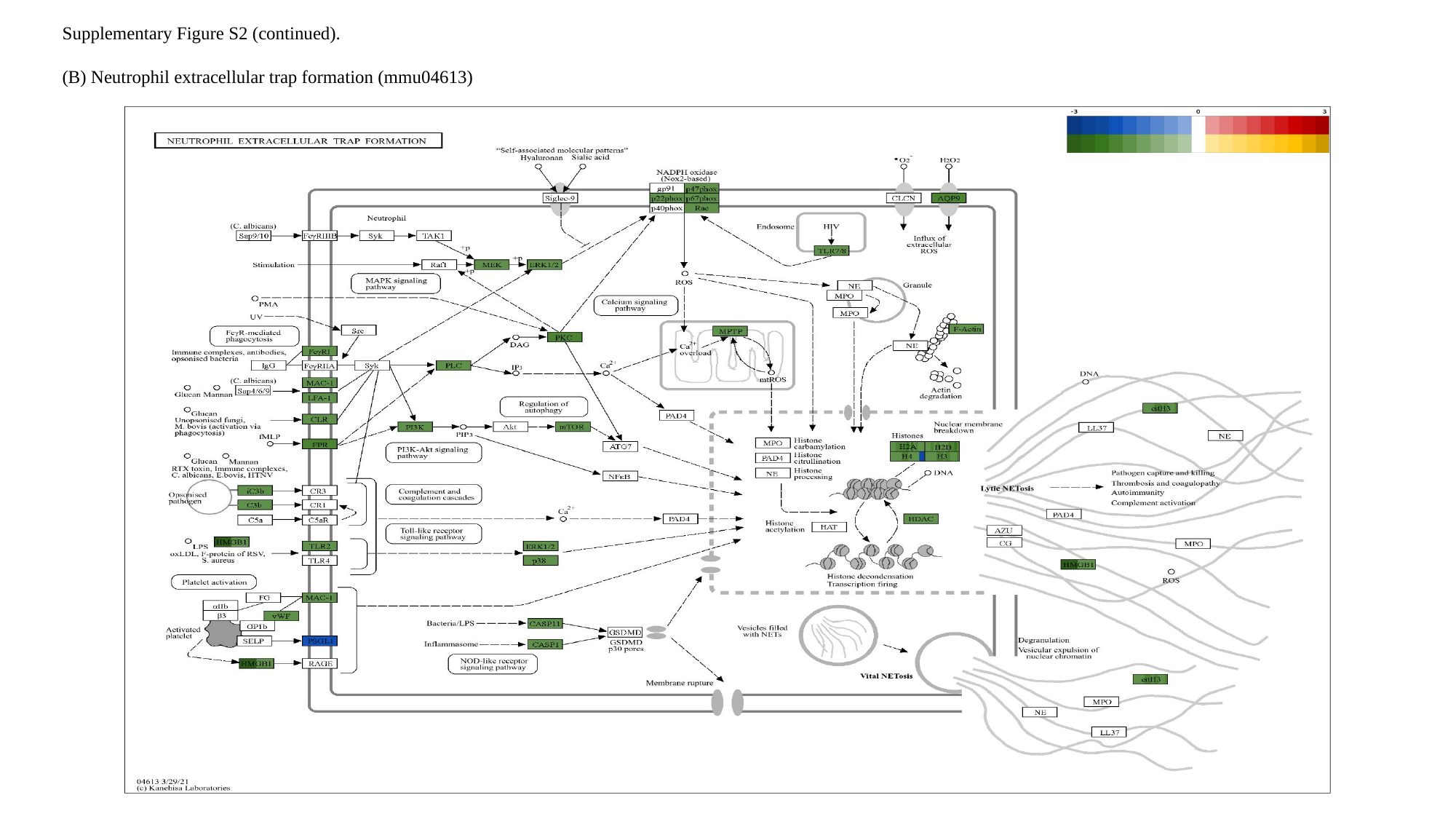

Supplementary Figure S2 (continued).
(B) Neutrophil extracellular trap formation (mmu04613)

## Slide 4
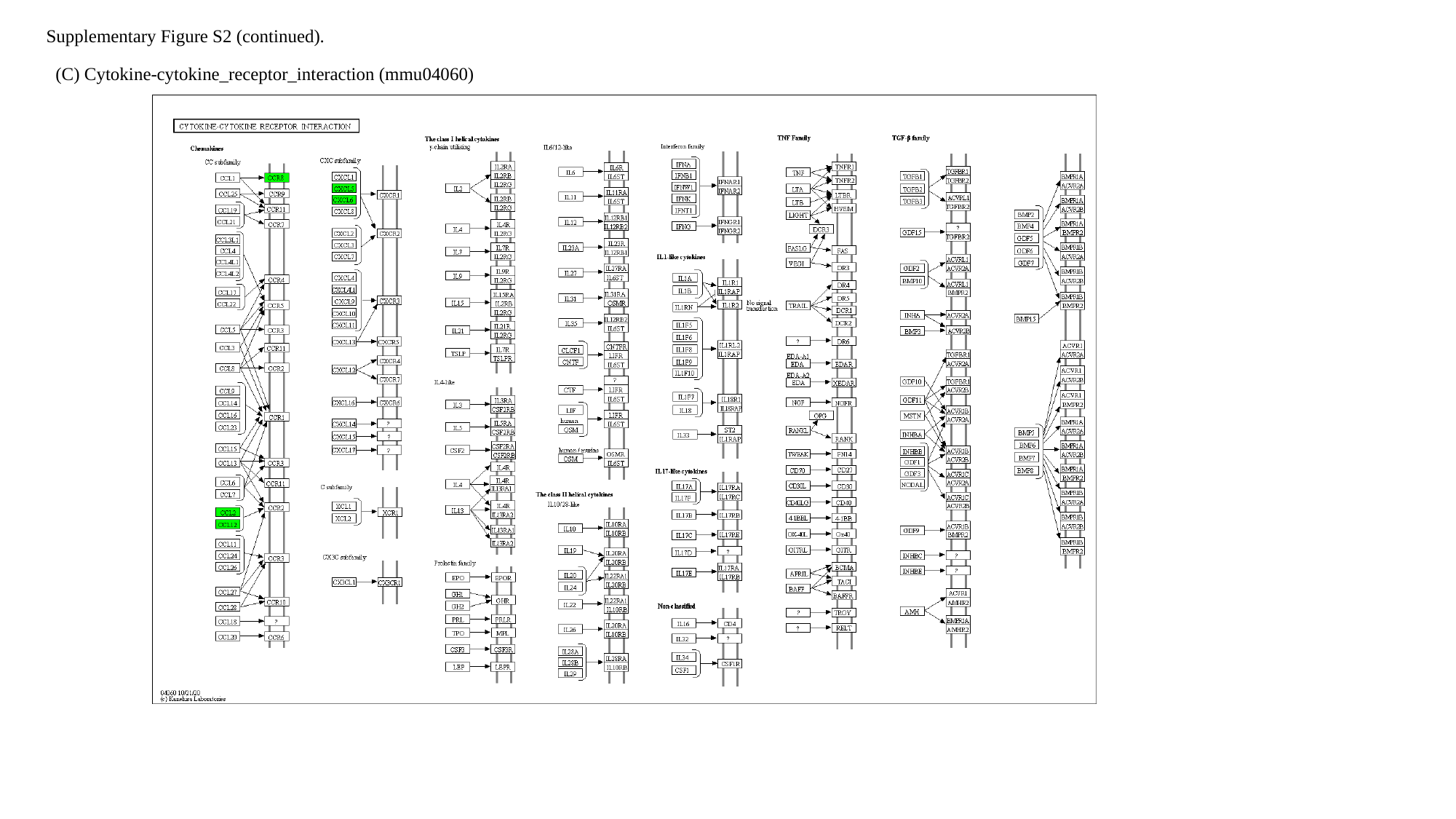

Supplementary Figure S2 (continued).
(C) Cytokine-cytokine_receptor_interaction (mmu04060)

## Slide 5
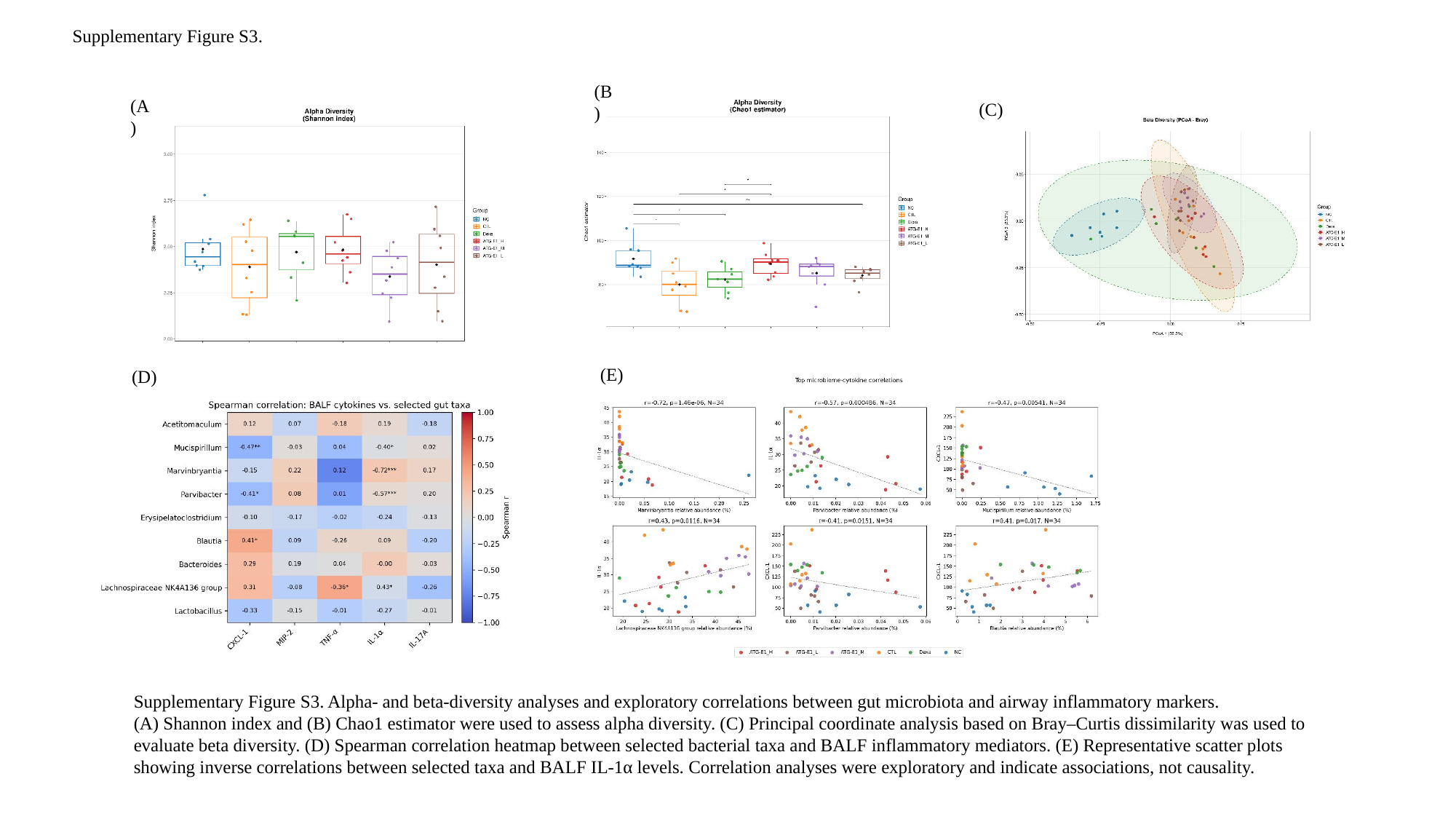

Supplementary Figure S3.
(B)
(A)
(C)
(E)
(D)
Supplementary Figure S3. Alpha- and beta-diversity analyses and exploratory correlations between gut microbiota and airway inflammatory markers.
(A) Shannon index and (B) Chao1 estimator were used to assess alpha diversity. (C) Principal coordinate analysis based on Bray–Curtis dissimilarity was used to evaluate beta diversity. (D) Spearman correlation heatmap between selected bacterial taxa and BALF inflammatory mediators. (E) Representative scatter plots showing inverse correlations between selected taxa and BALF IL-1α levels. Correlation analyses were exploratory and indicate associations, not causality.

## Slide 6
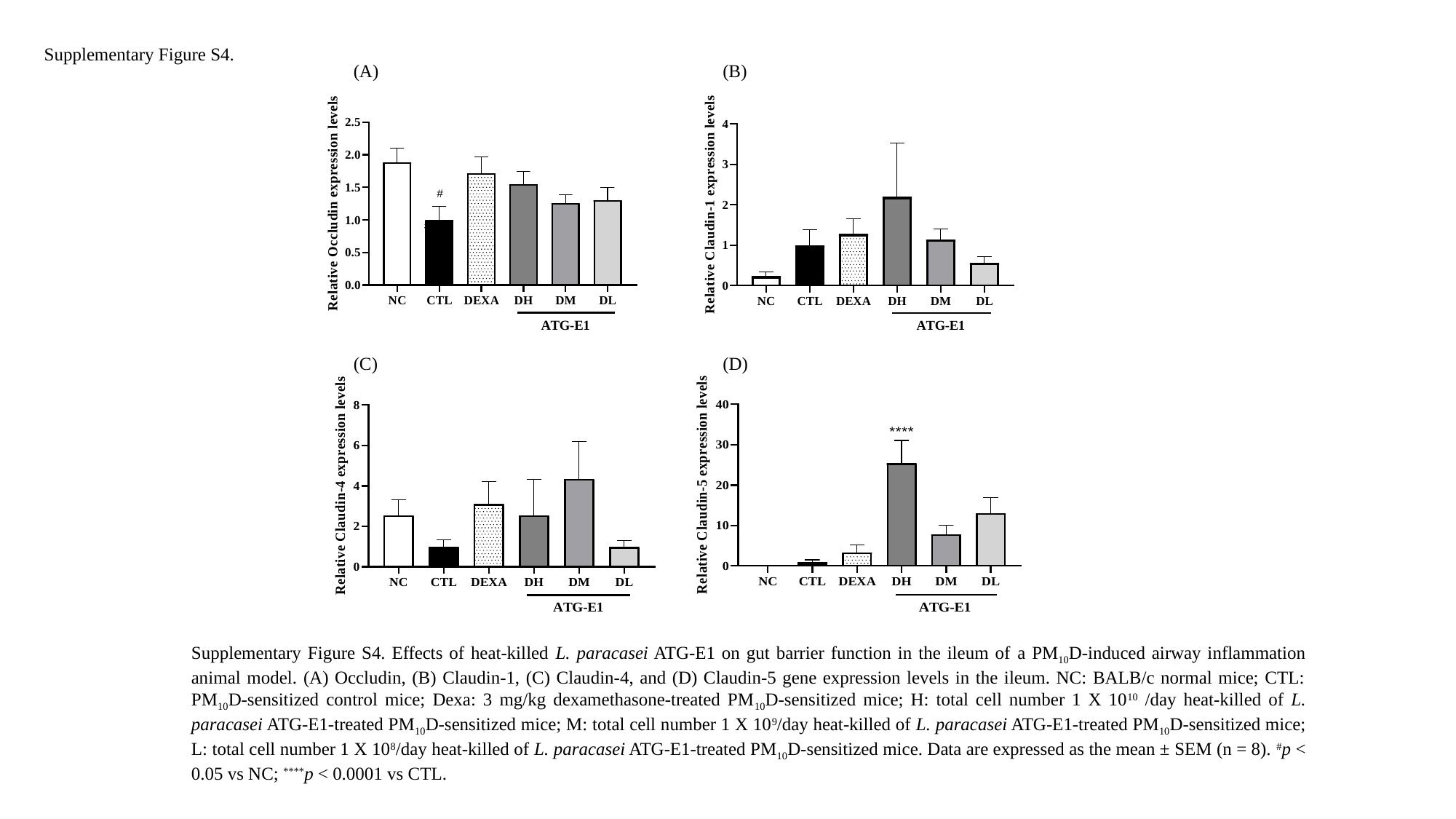

Supplementary Figure S4.
(A)
(B)
(C)
(D)
Supplementary Figure S4. Effects of heat-killed L. paracasei ATG-E1 on gut barrier function in the ileum of a PM10D-induced airway inflammation animal model. (A) Occludin, (B) Claudin-1, (C) Claudin-4, and (D) Claudin-5 gene expression levels in the ileum. NC: BALB/c normal mice; CTL: PM10D-sensitized control mice; Dexa: 3 mg/kg dexamethasone-treated PM10D-sensitized mice; H: total cell number 1 X 1010 /day heat-killed of L. paracasei ATG-E1-treated PM10D-sensitized mice; M: total cell number 1 X 109/day heat-killed of L. paracasei ATG-E1-treated PM10D-sensitized mice; L: total cell number 1 X 108/day heat-killed of L. paracasei ATG-E1-treated PM10D-sensitized mice. Data are expressed as the mean ± SEM (n = 8). #p < 0.05 vs NC; ****p < 0.0001 vs CTL.

## Slide 7
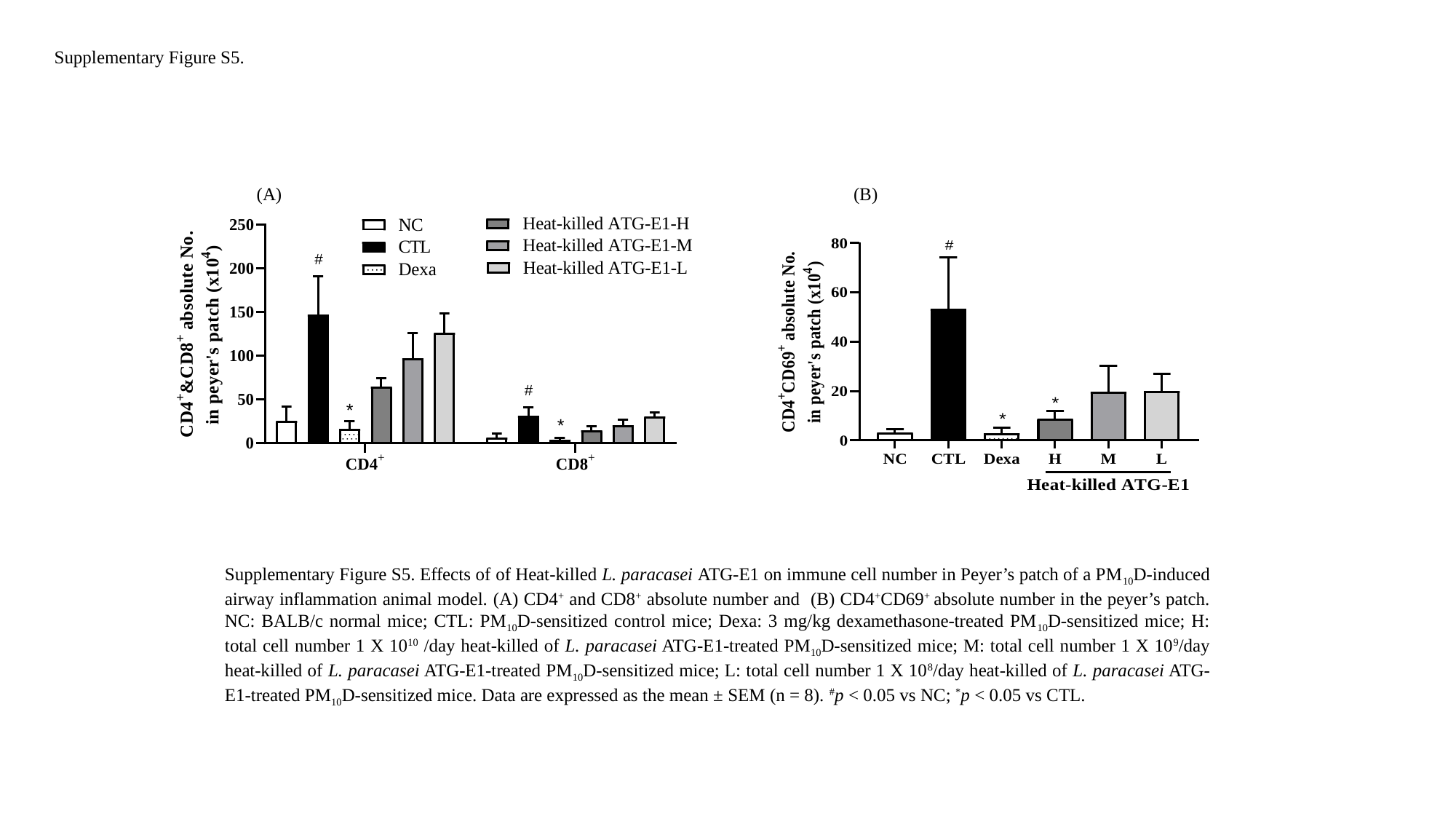

Supplementary Figure S5.
(A)
(B)
Supplementary Figure S5. Effects of of Heat-killed L. paracasei ATG-E1 on immune cell number in Peyer’s patch of a PM10D-induced airway inflammation animal model. (A) CD4+ and CD8+ absolute number and (B) CD4+CD69+ absolute number in the peyer’s patch. NC: BALB/c normal mice; CTL: PM10D-sensitized control mice; Dexa: 3 mg/kg dexamethasone-treated PM10D-sensitized mice; H: total cell number 1 X 1010 /day heat-killed of L. paracasei ATG-E1-treated PM10D-sensitized mice; M: total cell number 1 X 109/day heat-killed of L. paracasei ATG-E1-treated PM10D-sensitized mice; L: total cell number 1 X 108/day heat-killed of L. paracasei ATG-E1-treated PM10D-sensitized mice. Data are expressed as the mean ± SEM (n = 8). #p < 0.05 vs NC; *p < 0.05 vs CTL.

## Slide 8
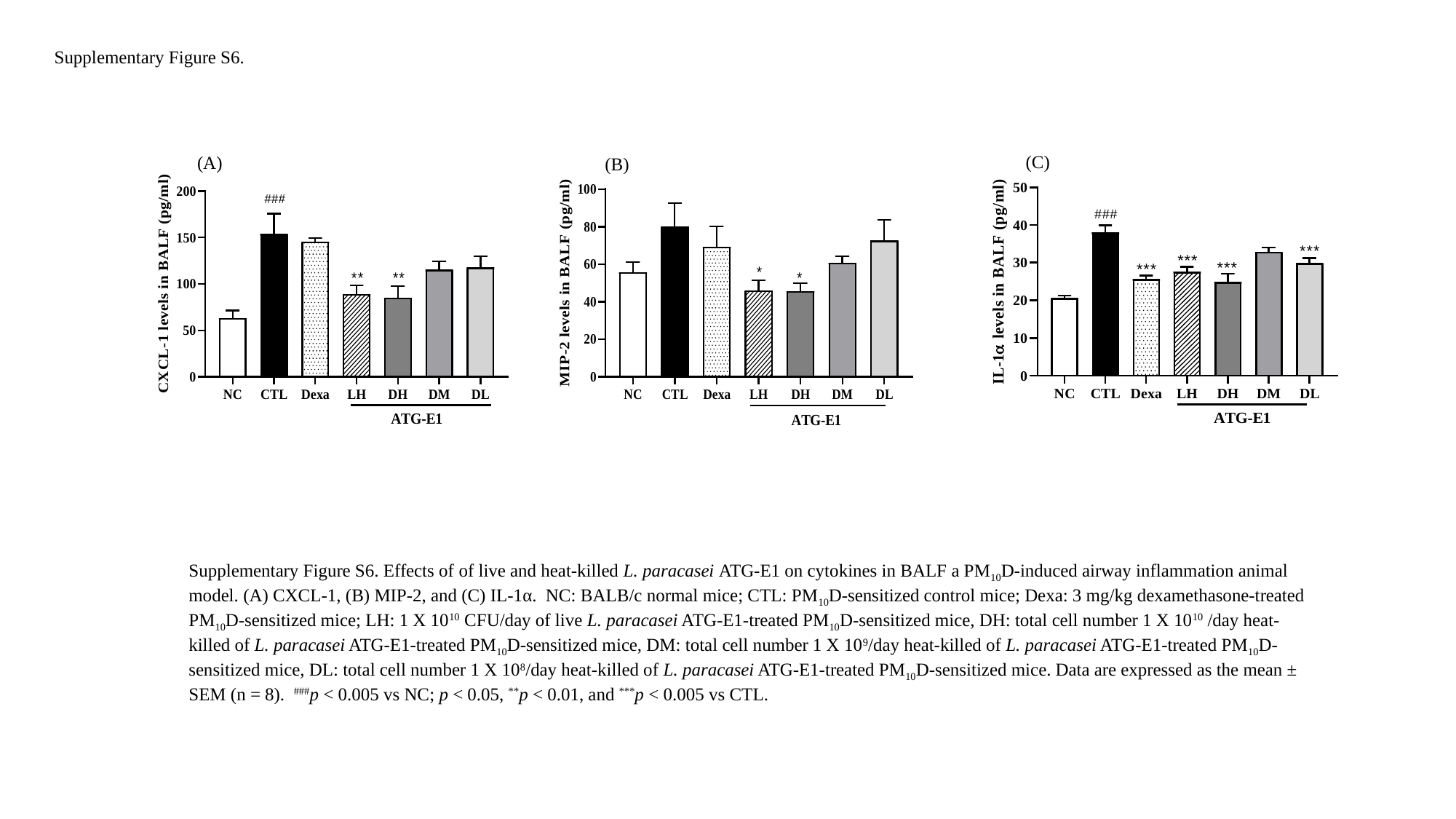

Supplementary Figure S6.
(C)
(A)
(B)
Supplementary Figure S6. Effects of of live and heat-killed L. paracasei ATG-E1 on cytokines in BALF a PM10D-induced airway inflammation animal model. (A) CXCL-1, (B) MIP-2, and (C) IL-1α. NC: BALB/c normal mice; CTL: PM10D-sensitized control mice; Dexa: 3 mg/kg dexamethasone-treated PM10D-sensitized mice; LH: 1 X 1010 CFU/day of live L. paracasei ATG-E1-treated PM10D-sensitized mice, DH: total cell number 1 X 1010 /day heat-killed of L. paracasei ATG-E1-treated PM10D-sensitized mice, DM: total cell number 1 X 109/day heat-killed of L. paracasei ATG-E1-treated PM10D-sensitized mice, DL: total cell number 1 X 108/day heat-killed of L. paracasei ATG-E1-treated PM10D-sensitized mice. Data are expressed as the mean ± SEM (n = 8). ###p < 0.005 vs NC; p < 0.05, **p < 0.01, and ***p < 0.005 vs CTL.

## Slide 9
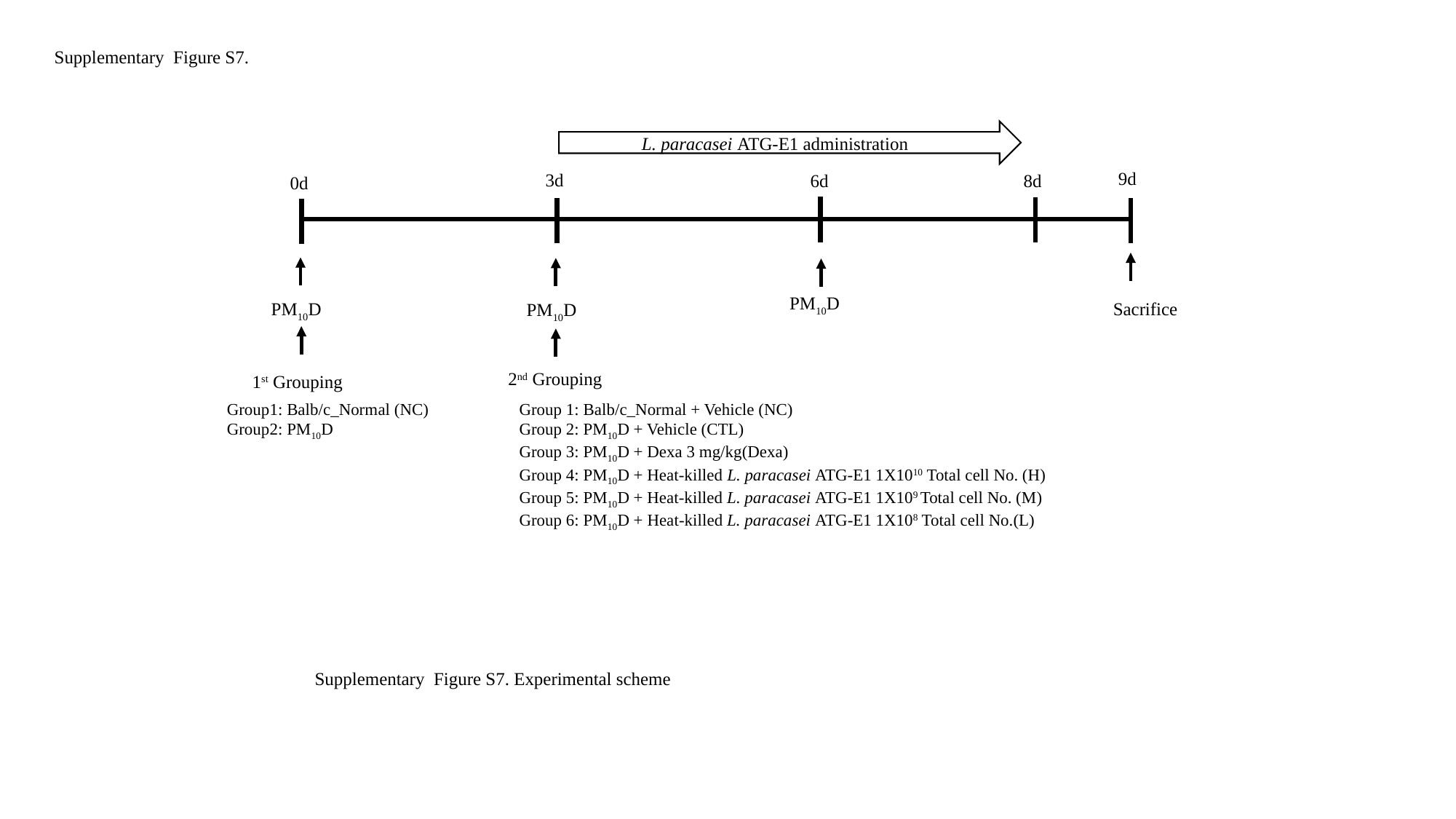

Supplementary Figure S7.
L. paracasei ATG-E1 administration
9d
3d
8d
6d
0d
PM10D
Sacrifice
PM10D
PM10D
2nd Grouping
1st Grouping
Group1: Balb/c_Normal (NC)
Group2: PM10D
Group 1: Balb/c_Normal + Vehicle (NC)
Group 2: PM10D + Vehicle (CTL)
Group 3: PM10D + Dexa 3 mg/kg(Dexa)
Group 4: PM10D + Heat-killed L. paracasei ATG-E1 1X1010 Total cell No. (H)
Group 5: PM10D + Heat-killed L. paracasei ATG-E1 1X109 Total cell No. (M)
Group 6: PM10D + Heat-killed L. paracasei ATG-E1 1X108 Total cell No.(L)
Supplementary Figure S7. Experimental scheme

## Slide 10
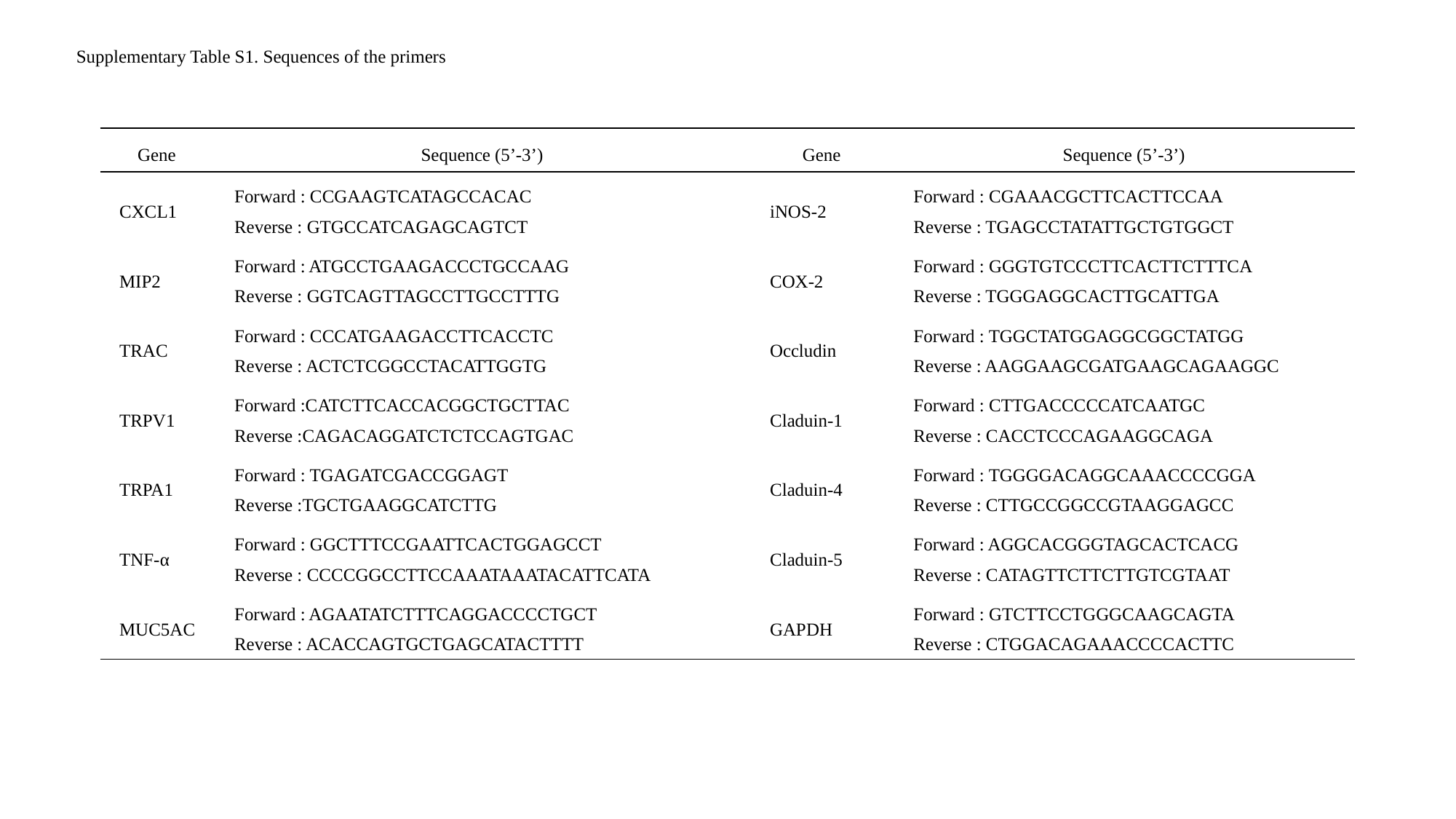

Supplementary Table S1. Sequences of the primers
| Gene | Sequence (5’-3’) | Gene | Sequence (5’-3’) |
| --- | --- | --- | --- |
| CXCL1 | Forward : CCGAAGTCATAGCCACAC Reverse : GTGCCATCAGAGCAGTCT | iNOS-2 | Forward : CGAAACGCTTCACTTCCAA Reverse : TGAGCCTATATTGCTGTGGCT |
| MIP2 | Forward : ATGCCTGAAGACCCTGCCAAG Reverse : GGTCAGTTAGCCTTGCCTTTG | COX-2 | Forward : GGGTGTCCCTTCACTTCTTTCA Reverse : TGGGAGGCACTTGCATTGA |
| TRAC | Forward : CCCATGAAGACCTTCACCTC Reverse : ACTCTCGGCCTACATTGGTG | Occludin | Forward : TGGCTATGGAGGCGGCTATGG Reverse : AAGGAAGCGATGAAGCAGAAGGC |
| TRPV1 | Forward :CATCTTCACCACGGCTGCTTAC Reverse :CAGACAGGATCTCTCCAGTGAC | Claduin-1 | Forward : CTTGACCCCCATCAATGC Reverse : CACCTCCCAGAAGGCAGA |
| TRPA1 | Forward : TGAGATCGACCGGAGT Reverse :TGCTGAAGGCATCTTG | Claduin-4 | Forward : TGGGGACAGGCAAACCCCGGA Reverse : CTTGCCGGCCGTAAGGAGCC |
| TNF-α | Forward : GGCTTTCCGAATTCACTGGAGCCT Reverse : CCCCGGCCTTCCAAATAAATACATTCATA | Claduin-5 | Forward : AGGCACGGGTAGCACTCACG Reverse : CATAGTTCTTCTTGTCGTAAT |
| MUC5AC | Forward : AGAATATCTTTCAGGACCCCTGCT Reverse : ACACCAGTGCTGAGCATACTTTT | GAPDH | Forward : GTCTTCCTGGGCAAGCAGTA Reverse : CTGGACAGAAACCCCACTTC |
