# Supplementary material for: Heat-Killed Lacticaseibacillus paracasei ATG-E1 Improves Particulate Matter 10 Plus Diesel Exhaust Particles (PM10D)-Induced Airway Inflammation
Source: Int J Mol Sci. 2026 Jul 1;27(13):5940. doi: 10.3390/ijms27135940 (PMC13361942; doi:10.3390/ijms27135940)
Supplement: Supplementary file 1 [file ijms-27-05940-s001.zip › Supplementary Materials and Methods.pdf]

Supplemental Methods

BALF collection and cytological analysis

To harvest the BALF, the lungs were lavaged once with DMEM and twice with PBS and centrifuged. The recovered supernatants were collected and stored at −80°C until determined for cytokines. The cell pellet was suspended in PBS and then used to analyze fluorescence-activated cell storing (FACS). The total cell numbers were counted using a hematocytometer. For cytological analysis, the cells collected from the BALF were stained with Diff-Quick Stain (Baxter Healthcare Corp., Miami, FL, USA), and the number of neutrophils was counted.

Digestion of the lung tissue and cell preparations

The lung tissue was minced and incubated in PBS containing collagenase IV (1 mg/mL) and dispase (2 mg/mL) at 37°C on a shaker. After incubation, the dissolved lung tissue was filtered using a cell strainer and centrifuged. The cell pellets were washed and suspended in PBS and then used to analyze FACS. The total cell numbers were determined using a hemocytometer.

Flow cytometry and gating strategy

To analyze the immune cell populations in BALF or lung tissues, flow cytometry was performed. Single-cell suspensions were initially gated to exclude debris based on forward scatter (FSC) and side scatter (SSC) profiles. Dead cells were excluded by gating out 7-AAD-positive cells. From the viable cell population, major leukocyte subsets—including

lymphocytes, neutrophils, and eosinophils—were identified according to their characteristic FSC and SSC properties. To further differentiate specific granulocyte populations, neutrophils were identified by gating the SiglecF^{-}/Gr-1^{+} population, which exhibits low expression of Siglec-F and high expression of Gr-1, as illustrated in the gating hierarchy.

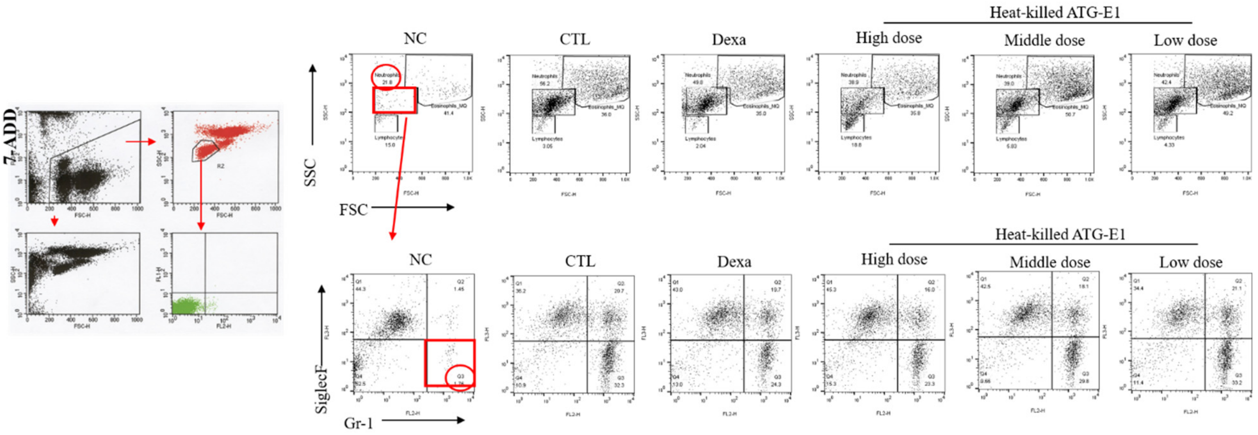

BALF cytological analysis

Cells were harvested and suspended in phosphate-buffered saline (PBS) at a density of 1x 10^5 cells/mL. A 100 µL aliquot of the cell suspension was loaded into each cytocentrifuge funnel assembly and spun onto glass slides using a cytocentrifuge (800 rpm, 5 min) at room temperature. After air-drying completely, the slides were stained using a commercial Diff-Quik stain kit according to the manufacturer's instructions. Briefly, the slides were sequentially dipped into Diff-Quik Fixative (methanol-based), Stain I (buffered eosin), and Stain II (buffered thiazine dye) 5 to 10 times each for 1 second per dip. Excess stains were drained, and the slides were rinsed gently with deionized water. After thoroughly air-drying, the slides were mounted with coverslip and examined under a light microscope.

Transcriptome analysis

Lung tissue samples were used for transcriptome analysis. For RNA-seq, each

sequencing sample consisted of pooled lung tissue from two mice. Three biological sequencing samples were prepared for each group included in the transcriptome comparison, including the normal control (NC), PM_{10}D-induced control (CTL), and heat-killed *L. paracasei* ATG-E1 high-dose-treated groups (H; 1×10^{10} total cells/day). Total RNA libraries were prepared using the TruSeq Stranded Total RNA LT Sample Prep Kit (Gold) and sequenced on an Illumina platform as paired-end 101-bp reads. The mouse reference genome mm10 and NCBI_108 annotation were used for alignment and gene annotation. Raw read quality was assessed using FastQC. Adapter sequences and low-quality bases were removed using Trimmomatic; bases with quality scores < 3 at read ends were trimmed, a sliding-window trimming step was applied with a window size of 4 and mean quality threshold of 15 and reads shorter than 36 bp were removed. After trimming, 59,513,850 ~ 80,795,786 processed reads were obtained per sample, with Q30 values of 95.36% ~ 95.96%. Cleaned reads were mapped to the mouse reference genome using HISAT2, and the overall mapping rate was 95.68% ~ 97.07%. Transcript assembly and expression quantification were performed using StringTie, and expression values were summarized as read counts, FPKM, and TPM. For differential gene-expression analysis, known-gene read-count data were used. Genes with zero counts in at least one of the 12 sequencing samples were excluded from statistical analysis; therefore, 20,103 genes among 45,777 detected genes were retained for downstream analysis. Read counts were normalized using the relative log expression (RLE) size-factor method in DESeq2. Differentially expressed genes (DEGs) were identified using the negative binomial Wald test in DESeq2. Genes satisfying |fold change| ≥ 2 and raw p-value < 0.05 were considered DEGs for downstream exploratory enrichment analysis

Reference

Yu, G., et al., 2012. clusterProfiler: an R package for comparing biological themes among gene clusters. Omics. 16**,** 284-7.

Altermann, E., Klaenhammer, T. R., 2005. PathwayVoyager: pathway mapping using the Kyoto Encyclopedia of Genes and Genomes (KEGG) database. BMC genomics. 6**,** 1.

Kanehisa, M., et al., 2016. KEGG: new perspectives on genomes, pathways, diseases and drugs. Nucleic acids research. 45**,** D353-D361.
